# A Mobile App–Based Gratitude Intervention’s Effect on Mental Well-Being in University Students: Randomized Controlled Trial

**DOI:** 10.2196/53850

**Published:** 2025-01-14

**Authors:** Chloë Fuller, Silvia Marin-Dragu, Ravishankar Subramani Iyer, Sandra Melanie Meier

**Affiliations:** 1 Department of Psychology, Saint Mary's University, Halifax, NS, Canada; 2 IWK Health Centre Department of Psychiatry & Specific Care Clinics, Department of Psychiatry, Dalhousie University, 5850/5980 University Ave, Halifax, NS, B3K 6R8, Canada, 1 902-470-7720; 3 Department of Psychology and Neuroscience, Dalhousie University, Halifax, NS, Canada; 4 Department of Computer Science, Dalhousie University, Halifax, NS, Canada

**Keywords:** gratitude intervention, smartphone app, gratitude exercises, psychological well-being, mobile phone

## Abstract

**Background:**

Gratitude interventions are used to cultivate a sense of gratitude for life and others. There have been mixed results of the efficacy of gratitude interventions’ effect on psychological well-being with a variety of populations and methodologies.

**Objectives:**

The objective of our study was to test the effectiveness of a gratitude intervention smartphone app on university students’ psychological well-being.

**Methods:**

We used a randomized experimental design to test our objective. Participants were recruited undergraduate students from a web-based university study recruitment system. Participants completed 90 web-based survey questions on their emotional well-being and personality traits at the beginning and end of the 3-week research period. Their depression, anxiety, and stress levels were measured with the Depression, Anxiety, and Stress Scale (DASS-21). After the baseline survey, participants were randomly assigned to either the control or the intervention. Participants in the intervention group used both a fully automated mobile sensing app and a gratitude intervention mobile iOS smartphone app designed for youth users and based on previous gratitude interventions and exercises. The gratitude intervention app prompted users to complete daily gratitude exercises on the app including a gratitude journal, a gratitude photo book, an imagine exercise, a speech exercise, and meditation. Participants in the control group used only the mobile sensing app, which passively collected smartphone sensory data on mobility, screen time, sleep, and social interactions.

**Results:**

A total of 120 participants met the inclusion criteria, and 27 were lost to follow-up for a total of 41 participants in the intervention group and 52 in the control group providing complete data. Based on clinical cutoffs from the baseline assessment, 56 out of 120 participants were identified as being in a subsample with at least moderate baseline symptomatology. Participants in the subsample with at least moderate baseline symptomatology reported significantly lower symptoms of depression, anxiety, and stress postintervention (Cohen *d*=−0.68; *P*=.04) but not in the full sample with low baseline symptomatology (Cohen *d*=0.16; *P*=.46). The number of times the app was accessed was not correlated with changes in either the subsample (*r*=0.01; *P*=.98) or the full sample (*r*=−0.04; *P*=.79).

**Conclusions:**

University students experiencing moderate to severe distress can benefit from a gratitude intervention smartphone app to improve symptoms of depression, anxiety, and stress. The number of times the gratitude intervention app was used is not related to well-being outcomes. Clinicians could look at incorporating gratitude apps with other mental health treatments or for those waitlisted as a cost-effective and minimally guided option for university students experiencing psychological distress.

## Introduction

### Background

Gratitude is the awareness of and appreciation for the positive aspects of life and the goodness of others, resulting in positive affect [1,2]. Gratitude has been shown to be an important resource for promoting and protecting mental well-being. Cross-sectional research has consistently reported that more grateful individuals are less depressed, anxious, and stressed than less grateful individuals [3]. Longitudinal studies in first-year undergraduate students further provided evidence that gratitude leads to improved well-being, with students higher in gratitude becoming less stressed, less depressed, and having higher perceived social support over the first term [4]. Gratitude is theorized to improve mental well-being through the mechanisms of positive affect (the positive emotion of gratitude has inherently a protective effect on various mental health problems) [1], positive outlook (grateful individuals’ cognitive schemas prompt them to interpret situations more positively) [5], coping strategies (grateful individuals make more positive coping appraisals) [6], and positive relationships (grateful individuals have broader skill repertoires helping them cultivate additional resources for support) [7]. In consequence, gratitude interventions have been developed to foster a sense of appreciation for life and others, yielding successful results and clinical relevance [1]. Gratitude interventions commonly consist of listing things to be grateful for, grateful contemplation, and behavioral expressions of gratitude [1]. Some exercises included in these interventions can be gratitude journals, gratitude letters, and practicing meditation and mindfulness. Gratitude journals prompt people to write about experiences that they are grateful for every day [8]. Gratitude letters are written expressions of gratitude to someone and their impact on the person’s life [9]. Gratitude interventions are valuable as they are easy to understand and complete. Furthermore, they can be done in various settings such as at home or virtually by a wide range of age groups [10]. They are flexible in their application and can be completed alongside other forms of psychotherapy or alone. In addition, they are generally enjoyed by those who engage in them [10]. Therefore, the accessibility of gratitude interventions makes them easy to integrate for widespread use by various populations.

### Effectiveness of Gratitude Interventions

Previous literature has suggested that gratitude interventions may help improve mental well-being by increasing gratitude, life satisfaction, and self-esteem while simultaneously decreasing negative affect, depression, anxiety, and stress. However, results have been mixed with effects varying by outcome, duration, follow-up length, format, and age. The first qualitative review investigating the efficacy of gratitude interventions included 12 studies and found that while a gratitude condition showed significant positive effects on mental well-being compared with a condition writing about daily hassles, no effects were observed in comparison with other control conditions [1]. Davis et al [10] re-evaluated the efficacy of gratitude interventions in 32 samples and found that gratitude interventions outperformed measurement-only control conditions for psychological well-being, but not for gratitude, suggesting overall weak efficacy of gratitude interventions compared with measurement-only controls. Renshaw and Olinger Steeves [11] examined gratitude interventions’ effect on youth by analyzing 6 studies and found that gratitude exercises to promote youth’s well-being and decrease distress were effective only for select outcomes and generally ineffective overall. Dickens [12] expanded on previous work with a series of meta-analyses on the effectiveness of gratitude interventions on unpooled outcome variables both postintervention and at a delayed follow-up. The results showed significant differences between control and intervention, providing evidence that gratitude interventions can positively impact people’s well-being, affect, and gratitude, as well as decreased depressive symptoms, with small to medium effect sizes both immediately and long term [12]. A recent systematic review on controlled trials of positive psychology digital interventions for youth and young adults found that these interventions enhance gratitude, hope, compassion, positive coping, and body image, with medium to large effects [13]. However, the results of a more recent meta-analysis including 27 studies focusing more specifically on symptoms of depression and anxiety suggest that there is a limited effect of gratitude interventions on reducing symptoms of depression and anxiety at postintervention and at follow-up [3].

### Web-Based and Mobile-Based Gratitude Interventions

Several recent studies have begun to explore the use of web-based gratitude interventions and gratitude apps. Web-based interventions can be delivered on a computer, smartphone, or other electronic devices, and are advantageous in that they are accessible, readily available, flexible in their use, and cost-effective; furthermore, they can be implemented into daily life, allow for monitoring engagement, and can encourage and improve consistent use [14-16]. A few studies have examined the effectiveness of gratitude apps on mental health in younger children [17], high school students [18], and adults [19]. Specifically, a study using a 6-week gratitude exercise app intervention found that intervention participants had significantly improved mental health outcomes compared with the control group, with effects continuing after the 6-week follow-up, which were not dependent on baseline well-being [19].

### Focus on Youth

Overall, previous literature indicates that gratitude interventions can effectively improve participants’ well-being. However, to our knowledge, no prior studies have conducted web-based gratitude interventions with a university-aged population. Furthermore, gratitude interventions have primarily focused on journaling and gratitude letters as forms of gratitude exercise. This study aims to address these gaps in the literature and expand the usage of gratitude interventions to a university student population, using diverse gratitude exercise modalities. University students experience higher stress levels than the rest of the population, making them an important population to provide mental health interventions [20]. Furthermore, youth mental health has been impacted by the COVID-19 pandemic, and university students have shown increased levels of depression, anxiety, and stress, as well as reported academic, health, and lifestyle concerns due to the pandemic [21]. Given the strong link between university students’ poor mental health [22] and the risk of low academic functioning and dropout [23], gratitude interventions might help mitigate not only mental health issues but also academic failure. Web-based interventions have proven to be generally effective, used, and liked by university students [24]. University students are less likely to seek help for psychological distress, but web-based interventions have been found to be a popular alternative to providing help and promoting well-being among students [25]. This is likely because web-based interventions are flexible, and university students can use them anywhere on their own time, which is advantageous with busy schedules or time constraints. Accordingly, university students, as an especially vulnerable population, might greatly benefit from a gratitude intervention app (GIA). If proven effective, the GIA could configure an easily accessible tool for university students to improve their mental well-being and academic performance.

### Research Question

Our study provides a 3-week gratitude intervention delivered through a smartphone app with 5 gratitude exercises to university students aged 18‐30 years to examine the intervention’s effects on depression, anxiety, and stress. This study will allow for a better understanding of a web-based GIA’s effect on psychological functioning and inform the use of this intervention for young adults and university students to support their psychological well-being.

### Hypotheses

We hypothesize the following:

1. Depression, anxiety, and stress symptoms will decrease from baseline to after the 3-week period in the group that practices the gratitude intervention tasks (intervention group) compared with the group that does not (control group).
2. The number of times the participants use the GIA will correlate with clinical symptomatology changes.

As many of the previous studies have focused on the general population with effectiveness depending on symptom severity [18], we explored our hypotheses in the full sample of university students and in a subsample of those who showed at least moderate symptomatology. Besides effectiveness, this study also investigated changes in potential mechanisms such as negative affectivity and social inhibition.

## Methods

### Participants

Participants were university students from Dalhousie University in Nova Scotia, Canada. They were recruited on the web through the Dalhousie SONA Experimental Participation System, a cloud-based research and participant management solution for universities. A priori power analyses were conducted using G*Power 5.0. A total sample size of 92 was estimated to be needed to achieve an 80% chance of detecting a small to medium effect size (*d*=0.25) at an α value of .05 for comparing the 2 groups at 2 time points. Anticipating approximately 30% attrition, we aimed to enroll a minimum of 120 participants.

The inclusion criteria were that participants must have been between ages of 18 and 30 years, spoke English, had access to a mobile phone with an iOS, had mobile phone and web-based literacy, and were not receiving treatment for any mental health condition. Participants also could not have any history of cardiovascular, endocrine, or kidney disease; tumor; hypertension; coarctation of the aorta; fibromuscular dysplasia; or sleep apnea to minimize possible confounding factors. A total of 137 participants met this inclusion criteria, and of those, 87.6% (120/137) enrolled and completed the baseline assessment.

### Design

This study used a randomized experimental design to test the effectiveness of a gratitude intervention smartphone app. Participants were asked to answer 90 questions in a web-based survey on emotional well-being and personality traits at the beginning and end of the 3-week research period. The intervention group used the GIA, and the Predicting Risk and Outcomes of Social Interactions (PROSIT) app, which is a mobile sensing app (for more details, see the study by MacLeod et al [26]). All participants used the PROSIT app in the background of their mobile devices throughout the 3-week study period. In addition, those allocated to the intervention group practiced gratitude intervention tasks during the same 3-week period using the GIA app.

### Procedure

The study took place between November 2021 and April 2023. Participants were recruited on the web through SONA Experimental Participation System at Dalhousie University. Individuals interested in registering for the study could sign up through SONA systems, which provided a link to the web-based consent form and study information on REDCap (Research Electronic Data Capture) [27]. REDCap is a free and secure application for web-based data collection approved by the Nova Scotia Health Authority and the Dalhousie University. Eligible participants who completed the consent form were sent a REDCap baseline assessment form on emotional well-being and personality traits through email. After completing the baseline measures, participants were randomly allocated to either the gratitude intervention group or the control group and were emailed instructions for downloading and using the iOS apps. Participants were randomized with a 1:1 allocation in blocks of 4 using a computer-generated algorithm. Randomization was conducted by a researcher who was not otherwise involved in the study. Data analysts had no interactions with participants. Participants in the gratitude intervention group downloaded and used both the PROSIT and GIA apps, while participants in the control group downloaded and used only the PROSIT app. Participants were instructed to complete the prompted gratitude exercise on the GIA app daily. After 3 weeks of consistent use of the apps, participants logged out of the apps and completed the outcome assessment form sent by email on REDCap. To improve adherence, participants using the GIA app received daily notifications via the app as well as a weekly reminder via email. Upon completion of the study, participants were further reimbursed through the assignment of US $21 Amazon gift card or 3 SONA points, which can be used for 3% extra credit toward a chosen university course final grade. Participants were also encouraged to complete questionnaires regardless of app usage.

### Gratitude Intervention App

The GIA app is an iOS smartphone app specifically designed for this study. The images of the GIA app can be found in Figures S1 and S2 in Multimedia Appendix 1. It is based on previous effective gratitude interventions and exercises [1,8,9], and its design and structure were specifically adapted for youth users. Users accessed it by logging in with an individualized username and password. The app consists of 5 different gratitude exercises: a gratitude journal, a photo book, an imagine exercise, a speech exercise, and meditation. Gratitude journals have been successfully used in previous gratitude intervention studies to encourage users to write a journal entry about what they are grateful for that day [8,26]. The photo book asks users to upload photographs of 5 things they are grateful for, such as people, things, or activities in their life, and encourages users to be mindful throughout the next week of other things they are grateful for to photograph and upload. Expressing gratitude through photography has previously been shown to improve well-being and decrease stress [26]. The imagine exercise asks users to take time to imagine 5 things they are most grateful for in their life and to explain the details as to why they are grateful for the listed thing and the aspects that make them happy. The speaking-out-loud exercise asks users to think about 5 things they are grateful for and talk about them for 90 seconds each, recording with the app. The meditation exercise prompts users to listen to a gratitude-based guided meditation recording and follow its instructions for being grateful in the moment and relaxing. Meditation has been used in previous gratitude interventions with significant effects on well-being and gratitude [26,28].

Every day during the 3-week study period, users received a notification from the app prompting them to complete the exercise for that day. The app is green and white in color, with an icon featured on the home and login pages of a flower with a heart rate design. Technical support was available through email for installation, usage, or login issues.

### Measurements

Participants’ demographic information was recorded at baseline via the secure REDCap survey application. Participants were asked to provide their age, biological sex, gender, sexual orientation, level of education, and race. Questionnaires measuring the outcome variables of interest (anxiety, depression, stress level, negative affectivity, and social inhibition) were assessed in the baseline and outcome assessment forms before and after the 3-week research period. They were automatically sent to participants via REDCap. PROSIT and GIA app usage (ie, registration date, frequency of accessing the app, and date of last visit) was logged.

### Outcome

Participant’s depression, anxiety, and stress levels were measured with the Depression, Anxiety, and Stress Scale (DASS-21) [29], which is a set of 3 self-report scales each consisting of 7 items to measure the individual’s emotional states of depression (“I couldn’t seem to experience any positive feeling at all”), anxiety (“I felt I was close to panic”), and stress (“I found it difficult to relax”). Participants rated how much each statement applied to them in the past week, from 0 (“Did not apply to me at all”) to 3 (“Applied to me very much or most of the time”). High scores indicate higher levels of symptoms of anxiety, depression, and stress. There are subscale score cutoffs for normal, mild, moderate, severe, and extremely severe emotional states. Participants were considered to show at least moderate symptomatology if they scored higher than 7 on the depression subscale, 6 on the anxiety subscale, and 10 on the stress subscale. The DASS-21 has shown good psychometric properties, with strong internal consistency in both total scoring and scale set scoring [30,31], and has been found to have adequate construct validity [29,31]. In the current sample, the reliability of DASS-21 was good with Cronbach α values of 0.89, 0.82, and 0.76 for the subscales of depression, anxiety, and stress, respectively.

### Mechanisms

Negative affectivity and social inhibition were measured with the Type D Personality Scale (DS14). The DS14 contains 14 items and consists of 2 subscales: negative affectivity (“I often make a fuss of unimportant things”) and social inhibition (“I find it hard to start a conversation”). Participants answered the items from 0 (“False’) to 4 (“True”), resulting in maximum scores of 28 for negative affectivity and social inhibition. The DS14 has shown good internal consistency for both negative affectivity and social inhibition [32]. In the current sample, the reliability of DS14 was good with Cronbach α values of 0.84 and 0.86 for the subscales of negative affectivity and social inhibition, respectively. All measures were completed on the web.

### Data Analysis

All statistical analyses were completed using R programming language (R version 4.2.2) and RStudio (version 2023.03.0+ Build 386). Descriptive statistics were computed for demographics and outcome measures at baseline in the full sample of students and a subsample of those who showed at least moderate symptomatology. Two-tailed independent sample *t* tests and *χ*² tests were used to test for any differences between the gratitude intervention group and the control group at baseline. We further compared participants who dropped out with those who completed the study on demographics, and baseline outcome measures, using independent sample *t* tests and *χ*² tests.

A longitudinal analysis of covariance, using linear mixed modeling, was conducted to test the effects of treatment on outcome and mechanism measures (DASS-21 and DS14) to test the effectiveness of the intervention. Treatment effects were tested by entering 2-way interactions between time and group assignment. An α value of .05 was used to determine statistical significance. Cohen *d* effect sizes were derived based on recommendations for linear mixed models [32], where 0.20, 0.50, and 0.80 were interpreted as indicating small, medium, and large effects, respectively [33]. An intent-to-treat approach was used, consistent with CONSORT guidelines (ie, all participants who were randomized to receive enrollment information for the intervention were included in analyses) [34]. Missing data were handled using maximum likelihood estimation [35]. To assess possible dose–response relationships in the gratitude intervention group, Spearman correlations were calculated between the number of times the app was used and changes in clinical symptomatology as measured by the DASS-21. To explore potential differences in technology engagement, a linear regression model compared the daily usage of the PROSIT app across groups.

### Ethical Considerations

The study was approved by the research ethics board committee of Dalhousie University in Halifax, Nova Scotia, Canada (REB#2021-5460). Before participation, all study participants were required to sign a primary informed consent form, thereby confirming their engagement in the survey process, app usage, future secondary data analysis, and the ability to opt out of the study without reason. The original consent covered secondary data analysis without additional consent. All study data were deidentified. Participants who completed the study were reimbursed with a US $21 Amazon gift card.

## Results

### Recruitment and Participant Characteristics

**Figure 1.**
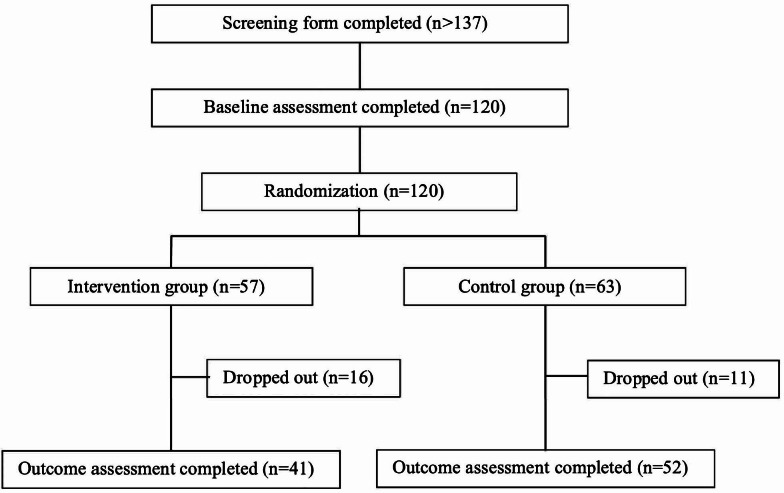
CONSORT (Consolidated Standards of Reporting Trails) flow diagram of the study.

Participant enrollment, allocation, and retention information are provided in line with the CONSORT (Consolidated Standards of Reporting Trails) flow diagram (Figure 1). Recruitment ran from November 11, 2021, to March 15, 2023. More than 137 individuals completed the eligibility screener for the study. Out of the 137 individuals who met the inclusion criteria, 87.6% (120/137) were enrolled and completed the baseline assessment. In the full sample, 57 participants were randomized to the gratitude intervention group and 63 to the control group of whom 31 and 25 displayed at least moderately significant symptomatology. Of those participants randomized to the gratitude intervention group, 77.2% (44/57) started and used the app in the full sample and 67.7% (21/31) in the subsample. Follow-ups were completed 3 weeks later. In the full sample, 28.1% (16/57) of gratitude intervention participants and 23.8% (15/63) of control participants were lost to follow-up. In the subsample 35.5% (11/31) of gratitude intervention participants and 24.0% (6/25) control participants were lost. These differences were not statistically significant (*P*=.59 in the full sample and *P*=.35 in the subsample).

Participant sociodemographic characteristics and baseline outcome measures are presented for the intervention group and the control group for both the full sample and the subsample showing at least moderate symptomatology (Multimedia Appendix 2). Participants were on average 19.8 years of age and the large majority (102/120, 85%) identified as female. Across both samples, most participants (117/120, 98% in the full sample and 54/56, 96% in the subsample) had completed some form of postsecondary education and most (86/117, 73% and 39/56, 70%) identified as being of European descent. Mean levels of depression, anxiety, and stress symptoms as measured by the DASS-21 were below the established clinical cutoffs at baseline for the full sample but not for the subsample. After randomization, there was no difference between the gratitude intervention group and the control group for the full sample and the subsample in any baseline sociodemographic characteristics and outcome measures.

### Dropout and Adherence

No statistically significant differences were observed in baseline characteristics between participants who completed the follow-up assessment and those lost to attrition for the full sample and the subsample. Furthermore, participants in the gratitude intervention group were no more likely to drop out than participants in the control group.

Log data showed that most participants (44/57, 77%) in the full sample randomized to the gratitude intervention group accessed the app. Participants opened the app on average 18 times (SD 11.04) over 9 days (SD 6.90). Participants in the subsample showing at least moderate symptomatology used the app slightly less, which was not statistically significant (*P*=.34); specifically, 68% (21/31) of participants accessed the app on average 16 times (SD 11.27) over 9 days (SD 7.73).

### Treatment Effects

The results of the mixed model analyses are shown in Table 1. There were no adverse events related to study participation. The results indicated a statistically significant medium treatment effect (ie, time × group interaction) for mental health symptoms (Cohen *d*=−0.68, 95% CI −1.34 to −0.03; *P*=.04) in the subsample showing at least moderate symptomatology but not in the full sample (*d*=0.16, 95% CI −0.26 to 0.58; *P*=.46). In the subsample, the gratitude intervention group scored significantly lower on depression, anxiety, and stress as measured by the DASS-21. The number of times the app was used was not correlated with changes in clinical symptomatology in the subsample with at least moderate symptomatology (*r*=0.01; *P*=.98) or in the full sample (*r*=−0.04; *P*=.79). The results indicated a statistically significant effect (ie, time × group interaction) for negative affectivity (*d*=−0.68, 95% CI −1.34 to −0.01; *P*=.046) but not for social inhibition (*d*=−0.61, 95% CI −1.27 to 0.05; *P*=.07) in the subsample showing at least moderate symptomatology. The gratitude intervention group scored significantly lower on negative affectivity and trendwise for social inhibition. No effects were observed in the full sample (negative affectivity: *d*=−0.18, 95% CI −0.60 to 0.24, *P*=.41; social inhibition: *d*=−0.18, 95% CI −0.60 to 0.24, *P*=.41). No differences in technology engagement as measured by the PROSIT app were observed in the gratitude intervention group compared with the control group in the subsample with at least moderate symptomatology (*t*_42_=0.56; *P*=.58) or in the full sample (*t*_100_=1.20; *P*=.23).

**Table 1.** Results of mixed model analyses.

	Value	SE	*t* value (*df*)		*P* value
Intercept	54.06	8.40	6.433 (54)		<.001
Baseline	8.76	8.39	1.045 (37)		.30
Treatment	−1.74	5.15	−0.338 (54)		.74
Baseline × treatment	−10.92	5.25	−2.081 (37)		.04

## Discussion

### Summary of Key Findings

Previous studies have examined the efficacy of gratitude interventions as a resource for psychological well-being with mixed results [1,10,12,14,18,36]. This study aimed to examine the effectiveness of a 3-week GIA on Canadian university students’ symptoms of depression, anxiety, and stress. We hypothesized that (1) initial depression, anxiety, and stress symptoms would decrease after the 3-week gratitude intervention period compared with the control group, in both the full sample and the subsample with at least moderate symptomatology, and (2) the number of times the participants used the GIA would be correlated with changes in clinical symptomatology in both the full sample and the moderate symptomatology subsample.

Our first hypothesis was partially supported. The mobile gratitude intervention was found to effectively improve mental health symptoms in the subsample showing at least moderate symptomatology, with those in the intervention group scoring significantly lower for symptoms of depression, anxiety, and stress after the 3-week intervention period than those in the subsample control group. However, these results were not found in the sample consisting of participants of any mental health symptomatology (full sample). These results indicate that university students with moderate and severe distress benefited from the intervention and highlight the suitability of gratitude interventions for this population. In contrast, our second hypothesis was not supported in both the subsample and the full sample as there was no correlation observed between the number of times the GIA was used and the changes in depression, anxiety, and stress symptoms. Interestingly, no differences between the gratitude intervention group and the control group were observed in technology engagement as measured using the PROSIT app. Thus, differences in motivation and comfortability in using technology are unlikely to explain the observed changes in mental health symptoms in the intervention group.

### Comparison With Other Research

The current gratitude intervention had a moderate effect size and exceeded previous effects found in meta-analyses of gratitude interventions [10,12,14]. The intervention had a similar effect size and ability to improve mental health outcomes as other recently conducted web-based gratitude interventions [18,37-39], although the current intervention examined here was probably less intense than those used in previous trials. This is of special interest as no additional guidance was provided in the current intervention, besides email-based technical support. Guidance can improve effectiveness [40,41] but it simultaneously reduces the feasibility of scaling the intervention into population-wide implementation. The current findings could indicate that guidance may be less necessary in highly educated digital natives such as university students resulting in higher scalability. Furthermore, our study revealed a similar effect size although it used an active control group (the mobile sensing app) as the comparison, instead of an inactive waitlist control group like previous trials, which can frequently lead to an overestimation of intervention effects [42].

The current gratitude intervention’s effect on mental health outcomes was dependent on participants’ baseline depression, anxiety, and stress, which contrasts previous findings [18]. Differences in the sample baseline well-being could explain this dissimilarity, as Bono et al [18] conducted their gratitude intervention with older participants of whom less than 20% experienced moderate symptoms of depression or anxiety due to the pandemic. This is in contrast to this study, in which nearly 50% of university students displayed moderate symptomatology. As such, the current findings show that gratitude interventions might be helpful not only for health promotion and illness prevention purposes but also for those who are clinically distressed thereby advancing current knowledge. Furthermore, the number of times the participants used the GIA was unrelated to well-being outcomes after the 3-week study period, contrasting previous studies [18,37]. This could be explained by the shorter intervention time frame compared with previous studies.

Besides symptomatology, the intervention also showed an effect on negative affectivity. These results are in line with previous work suggesting positive emotion as a potential underlying mechanism to explain the effect of gratitude on mental health [1,7,43]. In addition, a nonsignificant effect of the intervention on social inhibition was observed. Social support has previously been suggested as a process of change in gratitude interventions’ effects on mental well-being [1,2,7,37,43]. Yet other processes of change such as cognitive schemata and coping strategies were not assessed in this study [7,18,21,37,43] . Furthermore, this study describes only changes in potential mechanisms and thus does not establish them as mediating factors or their temporal precedence.

While this study did not include participants already receiving mental health treatment, future studies, interventions, and mental health treatments could look to incorporating gratitude interventions with other mental health treatments. The potential additive effect of gratitude interventions could be explored in combination with cognitive behavioral therapy or medication treatments. They could be offered as an interim approach for individuals on a waitlist as using the app may either reduce their symptoms or prevent them from getting worse. Gratitude interventions could also be used to maintain treatment success after discharge. Moreover, as the app targets gratitude rather than mental health problems, it may also help reduce the impact of stigma, which can prevent individuals from seeking support when distressed, and potentially increase engagement. Finally, because this intervention effectively improved mental health outcomes in university students with at least moderate baseline symptomatology, university health clinics could look to including mobile gratitude interventions as a cost-effective, minimally guided, and independent option for the promotion of student mental well-being, allowing more students to receive the help they are seeking. Specifically, students interested in self-driven mental well-being support may be encouraged to use gratitude interventions by counselors and other health care professionals. Gratitude could also be implemented in general university-led wellness programs.

### Strengths and Limitations

This is the first study to examine the effectiveness of a GIA in improving the mental well-being of university students in North America. Compared with previous gratitude interventions, the present intervention was also minimally guided in its delivery to participants [37], making it flexibly and independently used. The app also had various gratitude exercise options, allowing for different modalities of gratitude intervention on 1 interface.

Another strength of the gratitude intervention was that the mobile intervention app automatically recorded when participants used the exercises, providing data on when the app was used and allowing for uniquely objective insight into intervention utilization. Finally, another strength of the study was the exclusion of individuals already receiving mental health treatment, allowing for internal validity of the effectiveness of the intervention.

This study had several limitations. First, the study had a small sample size with a relatively homogenous sample population. The participants were predominantly Canadian, and female, and all were students. Thus, the study findings might not be generalizable to males. However, females are more vulnerable to depression and anxiety than males and are more likely to seek mental health treatment [42-44], making this form of intervention potentially more valuable and relevant to the female population. Furthermore, as previously discussed, university students are a population susceptible to stress and mental health issues [21], and web-based mental health interventions are effective and liked by this population [23,45]. However, future studies would benefit from examining the effects of mobile GIAs on less educated and more diverse samples by adapting different recruitment strategies (eg, by advertising in sports clubs or public transportation). Second, study dropout in the gratitude intervention group was relatively high and somewhat higher than in the control group. While this is a common finding in web-based intervention studies in university student populations (intervention group completion rates: 43%‐66%), this might limit the interpretation of results [46-48]. Reported trends could be partially related to specific participant characteristics (eg, having a stronger motivation for gratitude interventions) by the participants in the sample that adhered to the intervention. In addition, the app format was not compared with other forms of delivery and the effects of certain app features or persuasive elements were not tested. Further experiments are needed to unravel the specific effects of delivery features [49]. Third, there was no long-term follow-up on well-being outcomes after the intervention and follow-up occurred immediately only postintervention. Therefore, we are unable to conclude the long-term effects of mobile gratitude interventions in this population. Fourth, we investigated the gratitude intervention’s effect on the potential mechanisms of negative affectivity and social inhibition. Yet, this study did not have the power to examine them as mediators. In addition, more proximal mediators need to be considered for their effect on these more distal mechanisms. Future research should assess and test even more proximal mediators in serial multiple mediation models to further understand how the gratitude intervention works.

### Conclusions

This study is one of the first gratitude interventions delivered through an app to be used by a university student population. It demonstrates the effectiveness of a GIA in improving university students’ mental well-being by decreasing depression, anxiety, and stress symptoms after a 3-week intervention period. This study also found that app usage was not related to mental health outcomes, and intervention effectiveness was dependent on the participant’s baseline symptomatology. This app is valuable in its ability to be used virtually and independently, its potential to be used in conjunction with other mental health treatments, and its cost-effectiveness. Future studies should examine this intervention’s effectiveness in other populations and explore the unique impacts of different gratitude exercise modalities.

## Supplementary material

10.2196/53850 Multimedia Appendix 1 The gratitude intervention app.

10.2196/53850 Multimedia Appendix 2 Sociodemographic characteristics and outcome measures at baseline.

10.2196/53850 Checklist 1 CONSORT-EHEALTH (V 1.6.1).

## Abbreviations

DASS-21: Depression, Anxiety, and Stress Scale
DS14: Type D Personality Scale
GIA: gratitude intervention app
PROSIT: Predicting Risk and Outcomes of Social Interactions
REDCap: Research Electronic Data Capture
