# Supplementary material for: A Mobile App–Based Gratitude Intervention’s Effect on Mental Well-Being in University Students: Randomized Controlled Trial
Source: JMIR Mhealth Uhealth. 2025 Jan 14;13:e53850. doi: 10.2196/53850 (PMC11749078; doi:10.2196/53850)
Supplement: Multimedia Appendix 1 [file mhealth-v13-e53850-s001.docx]

This is a Multimedia Appendix to a full manuscript published in JMIR mHealth and uHealth. For full copyright and citation information see http://dx.doi.org/10.2196/jmir.53850

*Figure S1*. Landing and Activity Pages of the GIA App.

**
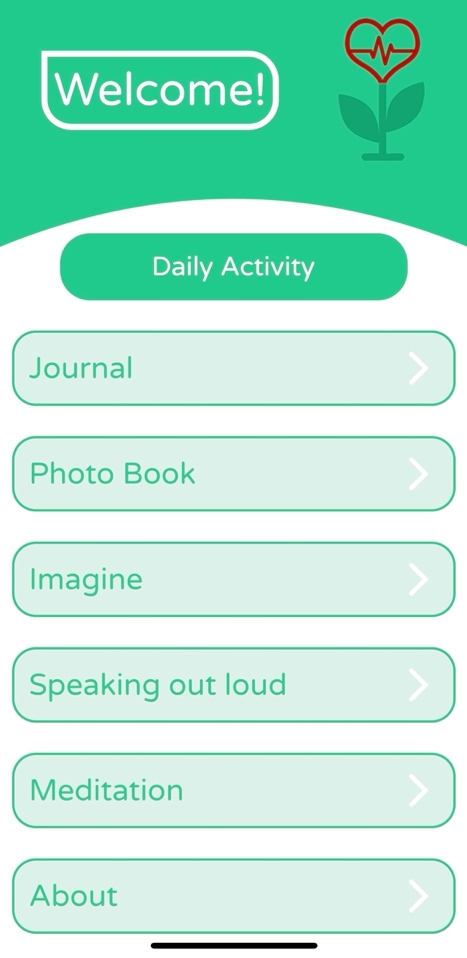

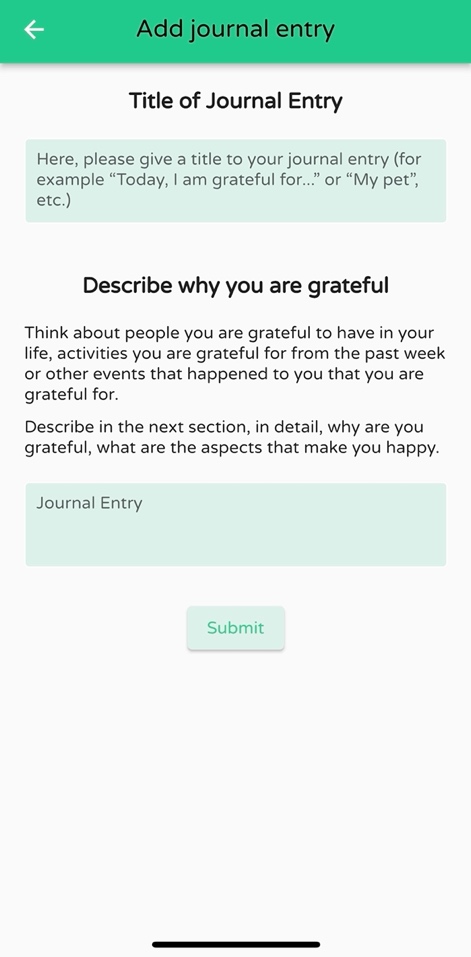

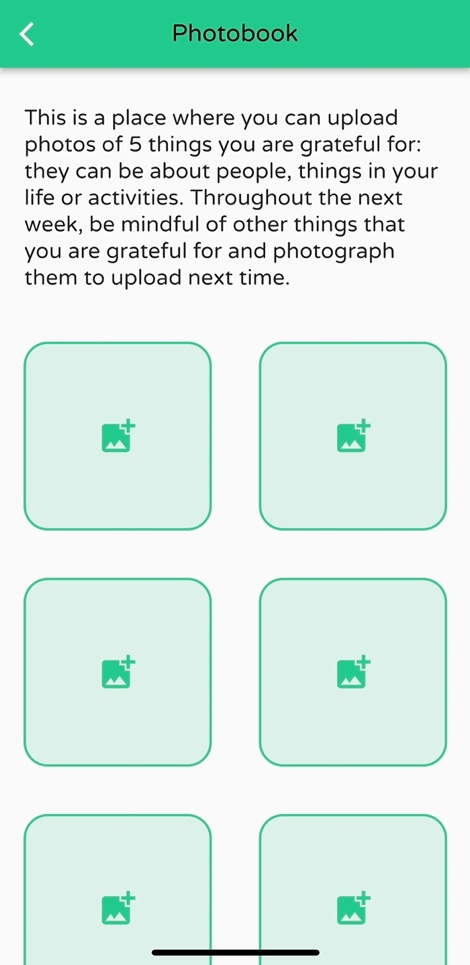
**

*Figure S2.* Screen layouts of the landing page of the GIA app, journal entry activity, photobook activity, imagine activity, speaking out loud activity, and meditation activity are displayed.

**
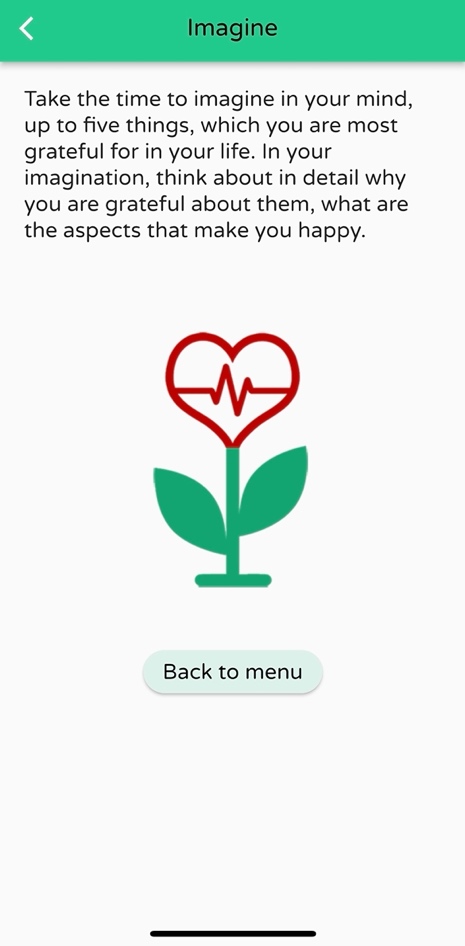

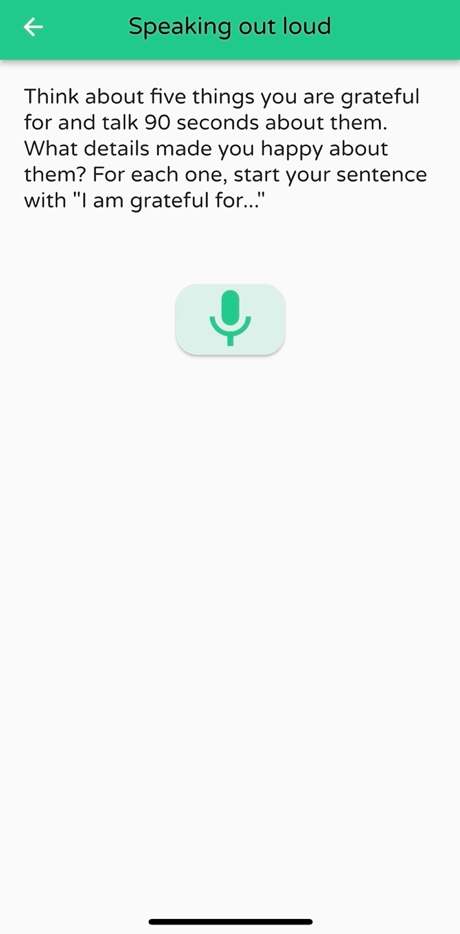

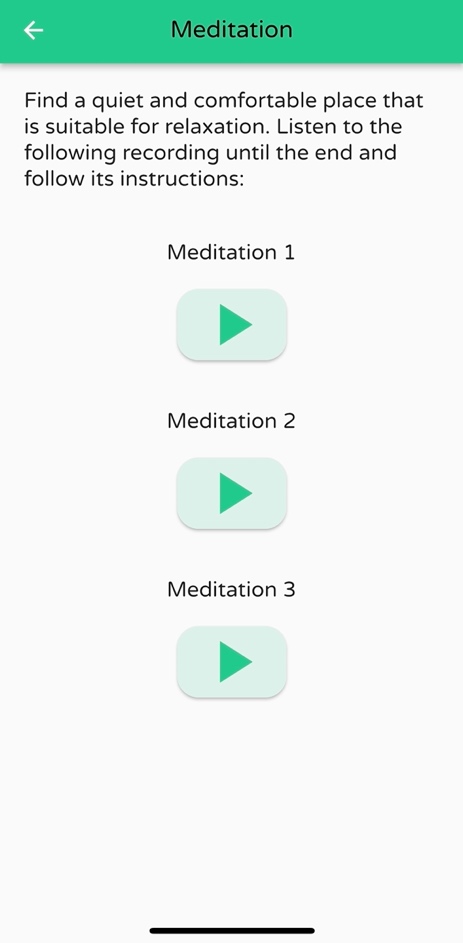
**
