# Supplementary material for: A Mobile App–Based Gratitude Intervention’s Effect on Mental Well-Being in University Students: Randomized Controlled Trial
Source: JMIR Mhealth Uhealth. 2025 Jan 14;13:e53850. doi: 10.2196/53850 (PMC11749078; doi:10.2196/53850)
Supplement: Multimedia Appendix 2 [file mhealth-v13-e53850-s002.docx]

This is a Multimedia Appendix to a full manuscript published in JMIR mHealth and uHealth. For full copyright and citation information see http://dx.doi.org/10.2196/jmir.53850

**Tables of Socio-demographic Characteristics for Full and Sub Samples**

Full Sample Socio-demographic Characteristics and Outcome Measures at Baseline.

|  | **Intervention (*n* =57)** | **Control (*n* =63)** | **Total (*n* =120)** | ***P*-value** |
| --- | --- | --- | --- | --- |
| **Age *m* (SD)** | 19.79 (1.5) | 19.8 (1.85) | 19.8 (1.68) | .95 |
| **Gender *n* (%)** | |  |  | .63 |
| Female | 47 (39) | 55 (46) | 102 (85) |  |
| Male | 10 (8) | 8 (7) | 18 (15) |  |
| **Education *n* (%)** | |  |  | .21 |
| Finished high school | 0 (0) | 3 (2) | 3 (2) |  |
| Postsecondary education | 57 (48) | 60 (50) | 117 (98) |  |
| **Ethnicity *n* (%)** | |  |  | .89 |
| Asian | 5 (4) | 11 (9) | 16 (13) |  |
| Black | 1 (1) | 4 (3) | 5 (4) |  |
| White | 40 (33) | 46 (38) | 86 (71) |  |
| Hispanic | 0 (0) | 4 (3) | 4 (3) |  |
| Indigenous | 3 (2) | 0 (0) | 3 (2) |  |
| Other | 13 (11) | 5 (4) | 18 (15) |  |
| **DASS21 *m* (SD)** | 35.72 (23.44) | 32.22 (20.32) | 33.88 (21.83) | .3989 |
| **DS14 *m* (SD)** | 25.39 (11.11) | 23.95 (10.38) | 24.63 (10.71) | .47 |

Subsample Socio-demographic Characteristics and Outcome Measures at Baseline.

|  | **Intervention (*n* =25)** | **Control (*n* =31)** | **Total (*n* =56)** | ***P*-value** |
| --- | --- | --- | --- | --- |
| **Age *m* (SD)** | 19.88 (1.92) | 19.94 (1.59) | 19.91 (1.73) |  |
| **Gender *n* (%)** | |  |  | .49 |
| Female | 24 (43) | 27 (48) | 51 (91) |  |
| Male | 1 (2) | 4 (7) | 5 (9) |  |
| **Education *n* (%)** | |  |  | .27 |
| Finished high school | 2 (4) | 0 (0) | 2 (4) |  |
| Postsecondary education | 23 (41) | 31 (55) | 54 (96) |  |
| **Ethnicity *n* (%)** | |  |  | .99 |
| Asian | 4 (7) | 3 (5) | 7 (12) |  |
| Black | 3 (5) | 1 (2) | 4 (7) |  |
| White | 17 (31) | 22 (39) | 39 (70) |  |
| Hispanic | 1 (2) | 0 (0) | 1 (2) |  |
| Indigenous | 0 (0) | 2 (4) | 2 (4) |  |
| Other | 4 (7) | 7 (12) | 11 (19) |  |
| **DASS21 *m* (SD)** | 42.32 (16.29) | 50.58 (21.44) | 51.36 (19.16) | .73 |
| **DS14 *m* (SD)** | 30.58 (9.62) | 30.04 (9.90) | 30.34 (9.66) | .84 |
