# Supplementary material for: A Mobile App–Based Gratitude Intervention’s Effect on Mental Well-Being in University Students: Randomized Controlled Trial
Source: JMIR Mhealth Uhealth. 2025 Jan 14;13:e53850. doi: 10.2196/53850 (PMC11749078; doi:10.2196/53850)
Supplement: Checklist 1 [file mhealth-v13-e53850-s003.pdf]

CONSORT-EHEALTH (V 1.6.1) - Submission/Publication Form

The CONSORT-EHEALTH checklist is intended for authors of randomized trials evaluating web-based and Internet-based applications/interventions, including mobile interventions, electronic games (incl multiplayer games), social media, certain telehealth applications, and other interactive and/or networked electronic applications. Some of the items (e.g. all subitems under item 5 - description of the intervention) may also be applicable for other study designs.

The goal of the CONSORT EHEALTH checklist and guideline is to be
a) a guide for reporting for authors of RCTs,
b) to form a basis for appraisal of an ehealth trial (in terms of validity)

CONSORT-EHEALTH items/subitems are MANDATORY reporting items for studies published in the Journal of Medical Internet Research and other journals / scientific societies endorsing the checklist.

Items numbered 1., 2., 3., 4a., 4b etc are original CONSORT or CONSORT-NPT (non-pharmacologic treatment) items.
Items with Roman numerals (i., ii, iii, iv etc.) are CONSORT-EHEALTH extensions/clarifications.

As the CONSORT-EHEALTH checklist is still considered in a formative stage, we would ask that you also RATE ON A SCALE OF 1-5 how important/useful you feel each item is FOR THE PURPOSE OF THE CHECKLIST and reporting guideline (optional).

Mandatory reporting items are marked with a red *.
In the textboxes, either copy & paste the relevant sections from your manuscript into this form - please include any quotes from your manuscript in QUOTATION MARKS,
or answer directly by providing additional information not in the manuscript, or elaborating on why the item was not relevant for this study.

YOUR ANSWERS WILL BE PUBLISHED AS A SUPPLEMENTARY FILE TO YOUR PUBLICATION IN JMIR AND ARE CONSIDERED PART OF YOUR PUBLICATION (IF ACCEPTED).
Please fill in these questions diligently. Information will not be copyedited, so please use proper spelling and grammar, use correct capitalization, and avoid abbreviations.

DO NOT FORGET TO SAVE AS PDF _AND_ CLICK THE SUBMIT BUTTON SO YOUR ANSWERS ARE IN OUR DATABASE !!!

Citation Suggestion (if you append the pdf as Appendix we suggest to cite this paper in the caption):
Eysenbach G, CONSORT-EHEALTH Group
CONSORT-EHEALTH: Improving and Standardizing Evaluation Reports of Web-based and Mobile Health Interventions
J Med Internet Res 2011;13(4):e126
URL: http://www.jmir.org/2011/4/e126/
doi: 10.2196/jmir.1923
PMID: 22209829

prositlab@gmail.com Switch accounts

Not shared

Draft saved

* Indicates required question

Your name *

First Last

Sandra Meier

Primary Affiliation (short), City, Country *

University of Toronto, Toronto, Canada

Dalhousie University, Halifax, Canada

Your e-mail address *

abc@gmail.com

prosit@dal.ca

Title of your manuscript *

Provide the (draft) title of your manuscript.

A Mobile Application-Based Gratitude Intervention's Effect on Mental Well-Being in University Students: Randomized Controlled Trial

Name of your App/Software/Intervention *

If there is a short and a long/alternate name, write the short name first and add the long name in brackets.

GIA (Gratitude Intervention App)

Evaluated Version (if any)

e.g. "V1", "Release 2017-03-01", "Version 2.0.27913"

Version 1.0.1, Release 2023-11-19

Language(s) *

What language is the intervention/app in? If multiple languages are available, separate by comma (e.g. "English, French")

English

URL of your Intervention Website or App

e.g. a direct link to the mobile app on app in appstore (itunes, Google Play), or URL of the website. If the intervention is a DVD or hardware, you can also link to an Amazon page.

This app was developed using TestFlight, which requires consistent updating and, therefore,

Must be a valid URL

URL of an image/screenshot (optional)

This app was developed using TestFlight, which requires consistent updating and, therefore,

Must be a valid URL

Accessibility *

Can an enduser access the intervention presently?

- ○ access is free and open
- ○ access only for special usergroups, not open
- ○ access is open to everyone, but requires payment/subscription/in-app purchases
- ◉ app/intervention no longer accessible
- ○ Other:

Primary Medical Indication/Disease/Condition *

e.g. "Stress", "Diabetes", or define the target group in brackets after the condition, e.g. "Autism (Parents of children with)", "Alzheimers (Informal Caregivers of)"

Anxiety, depression, stress

Primary Outcomes measured in trial *

comma-separated list of primary outcomes reported in the trial

stress, anxiety, depression

Secondary/other outcomes

Are there any other outcomes the intervention is expected to affect?

Your answer

Recommended "Dose" *

What do the instructions for users say on how often the app should be used?

- (selected) Approximately Daily
- Approximately Weekly
- Approximately Monthly
- Approximately Yearly
- "as needed"
- Other:

Approx. Percentage of Users (starters) still using the app as recommended after 3 months *

- ( ) unknown / not evaluated
- (x) 0-10%
- ( ) 11-20%
- ( ) 21-30%
- ( ) 31-40%
- ( ) 41-50%
- ( ) 51-60%
- ( ) 61-70%
- ( ) 71%-80%
- ( ) 81-90%
- ( ) 91-100%
- ( ) Other:

Overall, was the app/intervention effective? *

- ( ) yes: all primary outcomes were significantly better in intervention group vs control
- (x) partly: SOME primary outcomes were significantly better in intervention group vs control
- ( ) no statistically significant difference between control and intervention
- ( ) potentially harmful: control was significantly better than intervention in one or more outcomes
- ( ) inconclusive: more research is needed
- ( ) Other:

Article Preparation Status/Stage *

At which stage in your article preparation are you currently (at the time you fill in this form)

- ( ) not submitted yet - in early draft status
- ( ) not submitted yet - in late draft status, just before submission
- ( ) submitted to a journal but not reviewed yet
- (x) submitted to a journal and after receiving initial reviewer comments
- ( ) submitted to a journal and accepted, but not published yet
- ( ) published
- ( ) Other:

Journal *

If you already know where you will submit this paper (or if it is already submitted), please provide the journal name (if it is not JMIR, provide the journal name under "other")

- ( ) not submitted yet / unclear where I will submit this
- ( ) Journal of Medical Internet Research (JMIR)
- (x) JMIR mHealth and UHealth
- ( ) JMIR Serious Games
- ( ) JMIR Mental Health
- ( ) JMIR Public Health
- ( ) JMIR Formative Research
- ( ) Other JMIR sister journal
- ( ) Other:

Is this a full powered effectiveness trial or a pilot/feasibility trial? *

- ( ) Pilot/feasibility
- (x) Fully powered

Manuscript tracking number *

If this is a JMIR submission, please provide the manuscript tracking number under "other" (The ms tracking number can be found in the submission acknowledgement email, or when you login as author in JMIR. If the paper is already published in JMIR, then the ms tracking number is the four-digit number at the end of the DOI, to be found at the bottom of each published article in JMIR)

- ○ no ms number (yet) / not (yet) submitted to / published in JMIR
- ◉ Other: 53850

TITLE AND ABSTRACT

1a) TITLE: Identification as a randomized trial in the title

1a) Does your paper address CONSORT item 1a? *

I.e does the title contain the phrase "Randomized Controlled Trial"? (if not, explain the reason under "other")

- ◉ yes
- ○ Other:

1a-i) Identify the mode of delivery in the title

Identify the mode of delivery. Preferably use “web-based” and/or “mobile” and/or “electronic game” in the title. Avoid ambiguous terms like “online”, “virtual”, “interactive”. Use “Internet-based” only if Intervention includes non-web-based Internet components (e.g. email), use “computer-based” or “electronic” only if offline products are used. Use “virtual” only in the context of “virtual reality” (3-D worlds). Use “online” only in the context of “online support groups”. Complement or substitute product names with broader terms for the class of products (such as “mobile” or “smart phone” instead of “iphone”), especially if the application runs on different platforms.

	1	2	3	4	5	
subitem not at all important	○	○	○	○	◉	essential

Clear selection

Does your paper address subitem 1a-i? *

Copy and paste relevant sections from manuscript title (include quotes in quotation marks "like this" to indicate direct quotes from your manuscript), or elaborate on this item by providing additional information not in the ms, or briefly explain why the item is not applicable/relevant for your study

“A Mobile Application-Based Gratitude Intervention’s Effect on Mental Well-Being in University Students: Randomized Controlled Trial”

1a-ii) Non-web-based components or important co-interventions in title

Mention non-web-based components or important co-interventions in title, if any (e.g., “with telephone support”).

	1	2	3	4	5	
subitem not at all important	(•)	( )	( )	( )	( )	essential

Clear selection

Does your paper address subitem 1a-ii?

Copy and paste relevant sections from manuscript title (include quotes in quotation marks "like this" to indicate direct quotes from your manuscript), or elaborate on this item by providing additional information not in the ms, or briefly explain why the item is not applicable/relevant for your study

N/A, There were no non-web based components or important co-interventions.

1a-iii) Primary condition or target group in the title

Mention primary condition or target group in the title, if any (e.g., “for children with Type I Diabetes”) Example: A Web-based and Mobile Intervention with Telephone Support for Children with Type I Diabetes: Randomized Controlled Trial

	1	2	3	4	5	
subitem not at all important	( )	( )	( )	( )	(•)	essential

Clear selection

Does your paper address subitem 1a-iii? *

Copy and paste relevant sections from manuscript title (include quotes in quotation marks "like this" to indicate direct quotes from your manuscript), or elaborate on this item by providing additional information not in the ms, or briefly explain why the item is not applicable/relevant for your study

Yes, "University Students"

1b) ABSTRACT: Structured summary of trial design, methods, results, and conclusions

NPT extension: Description of experimental treatment, comparator, care providers, centers, and blinding status.

1b-i) Key features/functionalities/components of the intervention and comparator in the METHODS section of the ABSTRACT

Mention key features/functionalities/components of the intervention and comparator in the abstract. If possible, also mention theories and principles used for designing the site. Keep in mind the needs of systematic reviewers and indexers by including important synonyms. (Note: Only report in the abstract what the main paper is reporting. If this information is missing from the main body of text, consider adding it)

	1	2	3	4	5	
subitem not at all important	○	○	○	○	◉	essential

Clear selection

Does your paper address subitem 1b-i? *

Copy and paste relevant sections from the manuscript abstract (include quotes in quotation marks "like this" to indicate direct quotes from your manuscript), or elaborate on this item by providing additional information not in the ms, or briefly explain why the item is not applicable/relevant for your study

"Participants in the intervention group used both a fully automated mobile sensing app and a gratitude intervention mobile iOS smartphone app designed for youth users and based on previous gratitude interventions and exercises. The gratitude intervention app prompted users to complete daily gratitude exercises on the app including a gratitude journal, a gratitude photo book, an imagine exercise, a speech exercise, and meditation. Participants in the control group used only the mobile sensing app, which passively collected smartphone sensory data on mobility, screen time, sleep, and social interactions."

1b-ii) Level of human involvement in the METHODS section of the ABSTRACT

Clarify the level of human involvement in the abstract, e.g., use phrases like "fully automated" vs. "therapist/nurse/care provider/physician-assisted" (mention number and expertise of providers involved, if any). (Note: Only report in the abstract what the main paper is reporting. If this information is missing from the main body of text, consider adding it)

	1	2	3	4	5	
subitem not at all important	○	○	○	●	○	essential

Clear selection

Does your paper address subitem 1b-ii?

Copy and paste relevant sections from the manuscript abstract (include quotes in quotation marks "like this" to indicate direct quotes from your manuscript), or elaborate on this item by providing additional information not in the ms, or briefly explain why the item is not applicable/relevant for your study

"Participants in the intervention group used both a fully automated mobile sensing app and a gratitude intervention mobile iOS smartphone app designed for youth users and based on previous gratitude interventions and exercises."

1b-iii) Open vs. closed, web-based (self-assessment) vs. face-to-face assessments in the METHODS section of the ABSTRACT

Mention how participants were recruited (online vs. offline), e.g., from an open access website or from a clinic or a closed online user group (closed usergroup trial), and clarify if this was a purely web-based trial, or there were face-to-face components (as part of the intervention or for assessment). Clearly say if outcomes were self-assessed through questionnaires (as common in web-based trials). Note: In traditional offline trials, an open trial (open-label trial) is a type of clinical trial in which both the researchers and participants know which treatment is being administered. To avoid confusion, use “blinded” or “unblinded” to indicated the level of blinding instead of “open”, as “open” in web-based trials usually refers to “open access” (i.e. participants can self-enrol). (Note: Only report in the abstract what the main paper is reporting. If this information is missing from the main body of text, consider adding it)

	1	2	3	4	5	
subitem not at all important	○	○	○	◉	○	essential

Clear selection

Does your paper address subitem 1b-iii?

Copy and paste relevant sections from the manuscript abstract (include quotes in quotation marks "like this" to indicate direct quotes from your manuscript), or elaborate on this item by providing additional information not in the ms, or briefly explain why the item is not applicable/relevant for your study

"Participants were recruited undergraduate students from an online university study recruitment system". "Participants completed 90 online survey questions on their emotional well-being and personality traits at the beginning and end of the three-week research period."

1b-iv) RESULTS section in abstract must contain use data

Report number of participants enrolled/assessed in each group, the use/uptake of the intervention (e.g., attrition/adherence metrics, use over time, number of logins etc.), in addition to primary/secondary outcomes. (Note: Only report in the abstract what the main paper is reporting. If this information is missing from the main body of text, consider adding it)

	1	2	3	4	5	
subitem not at all important	○	○	○	○	◉	essential

Clear selection

Does your paper address subitem 1b-iv?

Copy and paste relevant sections from the manuscript abstract (include quotes in quotation marks "like this" to indicate direct quotes from your manuscript), or elaborate on this item by providing additional information not in the ms, or briefly explain why the item is not applicable/relevant for your study

Yes, "A total of 120 participants met the inclusion criteria, and 27 were lost to follow-up for a total of 41 participants in the intervention group and 52 in the control group providing complete data. Based on clinical cutoffs from the baseline assessment, 56 out of 120 participants were identified as being in a subsample with at least moderate baseline symptomatology."

1b-v) CONCLUSIONS/DISCUSSION in abstract for negative trials

Conclusions/Discussions in abstract for negative trials: Discuss the primary outcome - if the trial is negative (primary outcome not changed), and the intervention was not used, discuss whether negative results are attributable to lack of uptake and discuss reasons. (Note: Only report in the abstract what the main paper is reporting. If this information is missing from the main body of text, consider adding it)

	1	2	3	4	5	
subitem not at all important	○	○	○	○	◉	essential

Clear selection

Does your paper address subitem 1b-v?

Copy and paste relevant sections from the manuscript abstract (include quotes in quotation marks "like this" to indicate direct quotes from your manuscript), or elaborate on this item by providing additional information not in the ms, or briefly explain why the item is not applicable/relevant for your study

Yes, "University students experiencing moderate to severe distress can benefit from a gratitude intervention smartphone app to improve symptoms of depression, anxiety, and stress. The number of times the gratitude intervention app was used is not related to well-being outcomes."

INTRODUCTION

2a) In INTRODUCTION: Scientific background and explanation of rationale

2a-i) Problem and the type of system/solution

Describe the problem and the type of system/solution that is object of the study: intended as stand-alone intervention vs. incorporated in broader health care program? Intended for a particular patient population? Goals of the intervention, e.g., being more cost-effective to other interventions, replace or complement other solutions? (Note: Details about the intervention are provided in “Methods” under 5)

	1	2	3	4	5	
subitem not at all important	○	○	○	○	◉	essential

Clear selection

Does your paper address subitem 2a-i? *

Copy and paste relevant sections from the manuscript (include quotes in quotation marks "like this" to indicate direct quotes from your manuscript), or elaborate on this item by providing additional information not in the ms, or briefly explain why the item is not applicable/relevant for your study

"Overall, previous literature indicates that gratitude interventions can effectively improve participants' well-being. However, to our knowledge, no prior studies have conducted online gratitude interventions with a university-aged population."
"If proven effective the gratitude intervention app could configure an easily accessible tool for university students to improve their mental well-being and academic performance."

2a-ii) Scientific background, rationale: What is known about the (type of) system

Scientific background, rationale: What is known about the (type of) system that is the object of the study (be sure to discuss the use of similar systems for other conditions/diagnoses, if appropiate), motivation for the study, i.e. what are the reasons for and what is the context for this specific study, from which stakeholder viewpoint is the study performed, potential impact of findings [2]. Briefly justify the choice of the comparator.

	1	2	3	4	5	
subitem not at all important	○	○	○	●	○	essential

Clear selection

Does your paper address subitem 2a-ii? *

Copy and paste relevant sections from the manuscript (include quotes in quotation marks "like this" to indicate direct quotes from your manuscript), or elaborate on this item by providing additional information not in the ms, or briefly explain why the item is not applicable/relevant for your study

"Several recent studies have begun to explore the use of online gratitude interventions and gratitude apps. Online interventions can be delivered on a computer, smartphone, or other electronic devices, and are advantageous in that they are accessible, readily available, flexible in their use, and cost-effective; furthermore, they can be implemented into daily life, allow for monitoring engagement, and can encourage and improve consistent use [14-16]. A few studies have examined the effectiveness of gratitude apps on mental health in younger children [17], high school students [18], and adults [19]. Specifically, a study using a six-week gratitude exercise app intervention found that intervention participants had significantly improved mental health outcomes compared to the control group, with effects continuing after the six-week follow-up, which were not dependent on baseline well-being [19]."

2b) In INTRODUCTION: Specific objectives or hypotheses

Does your paper address CONSORT subitem 2b? *

Copy and paste relevant sections from the manuscript (include quotes in quotation marks "like this" to indicate direct quotes from your manuscript), or elaborate on this item by providing additional information not in the ms, or briefly explain why the item is not applicable/relevant for your study

"Our study provides a three-week gratitude intervention delivered through a smartphone app with five gratitude exercises to university students aged 18-30 years old, to examine the intervention's effects on depression, anxiety and stress. This study will allow for a better understanding of an online gratitude intervention app's effect on psychological functioning and inform the use of this intervention for young adults and university students to support their psychological well-being."

"1. Depression, anxiety, and stress symptoms will decrease from baseline to after the three-week period in the group that practices the gratitude intervention tasks (intervention group) compared to the group which does not (control group).
2. The number of times the participants use the gratitude intervention app will correlate with clinical symptomatology changes."

METHODS

3a) Description of trial design (such as parallel, factorial) including allocation ratio

Does your paper address CONSORT subitem 3a? *

Copy and paste relevant sections from the manuscript (include quotes in quotation marks "like this" to indicate direct quotes from your manuscript), or elaborate on this item by providing additional information not in the ms, or briefly explain why the item is not applicable/relevant for your study

"This study used a randomized experimental design to test the effectiveness of a gratitude intervention smartphone app."

3b) Important changes to methods after trial commencement (such as eligibility criteria), with reasons

Does your paper address CONSORT subitem 3b? *

Copy and paste relevant sections from the manuscript (include quotes in quotation marks "like this" to indicate direct quotes from your manuscript), or elaborate on this item by providing additional information not in the ms, or briefly explain why the item is not applicable/relevant for your study

NA - there were no changes to methods after trial commencement.

3b-i) Bug fixes, Downtimes, Content Changes

Bug fixes, Downtimes, Content Changes: ehealth systems are often dynamic systems. A description of changes to methods therefore also includes important changes made on the intervention or comparator during the trial (e.g., major bug fixes or changes in the functionality or content) (5-iii) and other “unexpected events” that may have influenced study design such as staff changes, system failures/downtimes, etc. [2].

	1	2	3	4	5	
subitem not at all important	◉	○	○	○	○	essential

Clear selection

Does your paper address subitem 3b-i?

Copy and paste relevant sections from the manuscript (include quotes in quotation marks "like this" to indicate direct quotes from your manuscript), or elaborate on this item by providing additional information not in the ms, or briefly explain why the item is not applicable/relevant for your study

NA – There were no bug fixes, downtimes, or content changes

4a) Eligibility criteria for participants

Does your paper address CONSORT subitem 4a? *

Copy and paste relevant sections from the manuscript (include quotes in quotation marks "like this" to indicate direct quotes from your manuscript), or elaborate on this item by providing additional information not in the ms, or briefly explain why the item is not applicable/relevant for your study

"The inclusion criteria were that participants must have been between ages 18 to 30, spoke English, had access to a mobile phone with an iPhone operating system (iOS), had mobile phone and internet literacy, and were not receiving treatment for any mental health condition. Participants also could not have any history of cardiovascular, endocrine, or kidney disease, tumour, hypertension, coarctation of the aorta, fibromuscular dysplasia, or sleep apnea, to minimize possible confounding factors."

4a-i) Computer / Internet literacy

Computer / Internet literacy is often an implicit “de facto” eligibility criterion - this should be explicitly clarified.

	1	2	3	4	5	
subitem not at all important	○	○	◉	○	○	essential

Clear selection

Does your paper address subitem 4a-i?

Copy and paste relevant sections from the manuscript (include quotes in quotation marks "like this" to indicate direct quotes from your manuscript), or elaborate on this item by providing additional information not in the ms, or briefly explain why the item is not applicable/relevant for your study

"The inclusion criteria were that participants must have been between ages 18 to 30, spoke English, had access to a mobile phone with an iPhone operating system (iOS), had mobile phone and internet literacy"

4a-ii) Open vs. closed, web-based vs. face-to-face assessments:

Open vs. closed, web-based vs. face-to-face assessments: Mention how participants were recruited (online vs. offline), e.g., from an open access website or from a clinic, and clarify if this was a purely web-based trial, or there were face-to-face components (as part of the intervention or for assessment), i.e., to what degree got the study team to know the participant. In online-only trials, clarify if participants were quasi-anonymous and whether having multiple identities was possible or whether technical or logistical measures (e.g., cookies, email confirmation, phone calls) were used to detect/prevent these.

	1	2	3	4	5	
subitem not at all important	○	○	○	◉	○	essential

Clear selection

Does your paper address subitem 4a-ii? *

Copy and paste relevant sections from the manuscript (include quotes in quotation marks "like this" to indicate direct quotes from your manuscript), or elaborate on this item by providing additional information not in the ms, or briefly explain why the item is not applicable/relevant for your study

"Participants were asked to answer 90 questions in an online survey"

4a-iii) Information giving during recruitment

Information given during recruitment. Specify how participants were briefed for recruitment and in the informed consent procedures (e.g., publish the informed consent documentation as appendix, see also item X26), as this information may have an effect on user self-selection, user expectation and may also bias results.

	1	2	3	4	5	
subitem not at all important	○	○	○	◉	○	essential

Clear selection

Does your paper address subitem 4a-iii?

Copy and paste relevant sections from the manuscript (include quotes in quotation marks "like this" to indicate direct quotes from your manuscript), or elaborate on this item by providing additional information not in the ms, or briefly explain why the item is not applicable/relevant for your study

"Individuals interested in registering for the study could sign up through SONA systems, which provided a link to the online consent form and study information on Research Electronic Data Capture (REDCap) [26]. REDCap is a free and secure application for online data collection approved by the Nova Scotia Health Authority and Dalhousie University. Eligible participants who completed the consent form were sent a REDCap baseline assessment form on emotional well-being and personality traits through email."

4b) Settings and locations where the data were collected

Does your paper address CONSORT subitem 4b? *

Copy and paste relevant sections from the manuscript (include quotes in quotation marks "like this" to indicate direct quotes from your manuscript), or elaborate on this item by providing additional information not in the ms, or briefly explain why the item is not applicable/relevant for your study

"Participants were recruited online through SONA Experimental Participation System at Dalhousie University."

4b-i) Report if outcomes were (self-)assessed through online questionnaires

Clearly report if outcomes were (self-)assessed through online questionnaires (as common in web-based trials) or otherwise.

	1	2	3	4	5	
subitem not at all important	○	○	○	●	○	essential

Clear selection

Does your paper address subitem 4b-i? *

Copy and paste relevant sections from the manuscript (include quotes in quotation marks "like this" to indicate direct quotes from your manuscript), or elaborate on this item by providing additional information not in the ms, or briefly explain why the item is not applicable/relevant for your study

"Participant's depression, anxiety, and stress levels were measured with the Depression, Anxiety, and Stress Scale (DASS-21) [30], which is a set of three self-report scales"

4b-ii) Report how institutional affiliations are displayed

Report how institutional affiliations are displayed to potential participants [on ehealth media], as affiliations with prestigious hospitals or universities may affect volunteer rates, use, and reactions with regards to an intervention.(Not a required item – describe only if this may bias results)

	1	2	3	4	5	
subitem not at all important	(selected)	○	○	○	○	essential

Clear selection

Does your paper address subitem 4b-ii?

Copy and paste relevant sections from the manuscript (include quotes in quotation marks "like this" to indicate direct quotes from your manuscript), or elaborate on this item by providing additional information not in the ms, or briefly explain why the item is not applicable/relevant for your study

NA – No institutional affiliations

5) The interventions for each group with sufficient details to allow replication, including how and when they were actually administered

5-i) Mention names, credential, affiliations of the developers, sponsors, and owners

Mention names, credential, affiliations of the developers, sponsors, and owners [6] (if authors/evaluators are owners or developer of the software, this needs to be declared in a "Conflict of interest" section or mentioned elsewhere in the manuscript).

	1	2	3	4	5	
subitem not at all important	○	○	○	○	●	essential

Clear selection

Does your paper address subitem 5-i?

Copy and paste relevant sections from the manuscript (include quotes in quotation marks "like this" to indicate direct quotes from your manuscript), or elaborate on this item by providing additional information not in the ms, or briefly explain why the item is not applicable/relevant for your study

Silvia Marin-Dragu B.A. in Computer Science and Psychology
Ravishankar Subramani Iyer M.A. in Computer Science
Sandra Meier, Ph.D. in Human Sciences

5-ii) Describe the history/development process

Describe the history/development process of the application and previous formative evaluations (e.g., focus groups, usability testing), as these will have an impact on adoption/use rates and help with interpreting results.

	1	2	3	4	5	
subitem not at all important	○	○	●	○	○	essential

Clear selection

Does your paper address subitem 5-ii?

Copy and paste relevant sections from the manuscript (include quotes in quotation marks "like this" to indicate direct quotes from your manuscript), or elaborate on this item by providing additional information not in the ms, or briefly explain why the item is not applicable/relevant for your study

The app was designed and piloted in-house in the PROSIT lab.

5-iii) Revisions and updating

Revisions and updating. Clearly mention the date and/or version number of the application/intervention (and comparator, if applicable) evaluated, or describe whether the intervention underwent major changes during the evaluation process, or whether the development and/or content was “frozen” during the trial. Describe dynamic components such as news feeds or changing content which may have an impact on the replicability of the intervention (for unexpected events see item 3b).

	1	2	3	4	5	
subitem not at all important	(●)	( )	( )	( )	( )	essential

Clear selection

Does your paper address subitem 5-iii?

Copy and paste relevant sections from the manuscript (include quotes in quotation marks "like this" to indicate direct quotes from your manuscript), or elaborate on this item by providing additional information not in the ms, or briefly explain why the item is not applicable/relevant for your study

NA – There were no app revisions or updates during the evaluation process.

5-iv) Quality assurance methods

Provide information on quality assurance methods to ensure accuracy and quality of information provided [1], if applicable.

	1	2	3	4	5	
subitem not at all important	(x)	( )	( )	( )	( )	essential

Clear selection

Does your paper address subitem 5-iv?

Copy and paste relevant sections from the manuscript (include quotes in quotation marks "like this" to indicate direct quotes from your manuscript), or elaborate on this item by providing additional information not in the ms, or briefly explain why the item is not applicable/relevant for your study

NA – There were no quality assurance methods implemented.

5-v) Ensure replicability by publishing the source code, and/or providing screenshots/screen-capture video, and/or providing flowcharts of the algorithms used

Ensure replicability by publishing the source code, and/or providing screenshots/screen-capture video, and/or providing flowcharts of the algorithms used. Replicability (i.e., other researchers should in principle be able to replicate the study) is a hallmark of scientific reporting.

	1	2	3	4	5	
subitem not at all important	○	○	○	◉	○	essential

Clear selection

Does your paper address subitem 5-v?

Copy and paste relevant sections from the manuscript (include quotes in quotation marks "like this" to indicate direct quotes from your manuscript), or elaborate on this item by providing additional information not in the ms, or briefly explain why the item is not applicable/relevant for your study

Screenshots of all pages of the app are included in the paper in the multimedia appendix.

5-vi) Digital preservation

Digital preservation: Provide the URL of the application, but as the intervention is likely to change or disappear over the course of the years; also make sure the intervention is archived (Internet Archive, webcitation.org, and/or publishing the source code or screenshots/videos alongside the article). As pages behind login screens cannot be archived, consider creating demo pages which are accessible without login.

	1	2	3	4	5	
subitem not at all important	○	○	○	◉	○	essential

Clear selection

Does your paper address subitem 5-vi?

Copy and paste relevant sections from the manuscript (include quotes in quotation marks "like this" to indicate direct quotes from your manuscript), or elaborate on this item by providing additional information not in the ms, or briefly explain why the item is not applicable/relevant for your study

http://prosit.meierlab.info/gratitude-intervention.html

5-vii) Access

Access: Describe how participants accessed the application, in what setting/context, if they had to pay (or were paid) or not, whether they had to be a member of specific group. If known, describe how participants obtained “access to the platform and Internet” [1]. To ensure access for editors/reviewers/readers, consider to provide a “backdoor” login account or demo mode for reviewers/readers to explore the application (also important for archiving purposes, see vi).

	1	2	3	4	5	
subitem not at all important	○	○	○	○	◉	essential

Clear selection

Does your paper address subitem 5-vii? *

Copy and paste relevant sections from the manuscript (include quotes in quotation marks "like this" to indicate direct quotes from your manuscript), or elaborate on this item by providing additional information not in the ms, or briefly explain why the item is not applicable/relevant for your study

"Users accessed it by logging in with an individualized username and password."

5-viii) Mode of delivery, features/functionalities/components of the intervention and comparator, and the theoretical framework

Describe mode of delivery, features/functionalities/components of the intervention and comparator, and the theoretical framework [6] used to design them (instructional strategy [1], behaviour change techniques, persuasive features, etc., see e.g., [7, 8] for terminology). This includes an in-depth description of the content (including where it is coming from and who developed it) [1],” whether [and how] it is tailored to individual circumstances and allows users to track their progress and receive feedback” [6]. This also includes a description of communication delivery channels and – if computer-mediated communication is a component – whether communication was synchronous or asynchronous [6]. It also includes information on presentation strategies [1], including page design principles, average amount of text on pages, presence of hyperlinks to other resources, etc. [1].

	1	2	3	4	5	
subitem not at all important	○	○	○	◉	○	essential

Clear selection

Does your paper address subitem 5-viii? *

Copy and paste relevant sections from the manuscript (include quotes in quotation marks "like this" to indicate direct quotes from your manuscript), or elaborate on this item by providing additional information not in the ms, or briefly explain why the item is not applicable/relevant for your study

Mode of Delivery, features/functionalities/components: “This study used a randomized experimental design to test the effectiveness of a gratitude intervention smartphone app. Participants were asked to answer 90 questions in an online survey on emotional well-being and personality traits at the beginning and end of the three-week research period. The intervention group used the Gratitude Intervention App (GIA), and the Predicting Risk and Outcomes of Social InTerActions (PROSIT) app, which is a mobile sensing app (for more details see [25]). All participants used the PROSIT app in the background of their mobile devices throughout the three-week study period. In addition, those allocated to the intervention group practiced gratitude intervention tasks during the same three-week period using the GIA app.”

More is found under the section ‘Gratitude Intervention App’ which details all of the components of the intervention.

Theoretical framework: The theoretical framework is described in the Background section of the introduction. It is also stated in the methods that “The GIA app is an iOS smartphone application specifically designed for this study. It is based on previous effective gratitude interventions and exercises, and its design and structure were specifically adapted for youth users.”

5-ix) Describe use parameters

Describe use parameters (e.g., intended “doses” and optimal timing for use). Clarify what instructions or recommendations were given to the user, e.g., regarding timing, frequency, heaviness of use, if any, or was the intervention used ad libitum.

	1	2	3	4	5	
subitem not at all important	○	○	○	○	●	essential

Clear selection

Does your paper address subitem 5-ix?

Copy and paste relevant sections from the manuscript (include quotes in quotation marks "like this" to indicate direct quotes from your manuscript), or elaborate on this item by providing additional information not in the ms, or briefly explain why the item is not applicable/relevant for your study

"Every day during the three-week study period, users received a notification from the app prompting them to complete the exercise for that day."

5-x) Clarify the level of human involvement

Clarify the level of human involvement (care providers or health professionals, also technical assistance) in the e-intervention or as co-intervention (detail number and expertise of professionals involved, if any, as well as “type of assistance offered, the timing and frequency of the support, how it is initiated, and the medium by which the assistance is delivered”. It may be necessary to distinguish between the level of human involvement required for the trial, and the level of human involvement required for a routine application outside of a RCT setting (discuss under item 21 – generalizability).

	1	2	3	4	5	
subitem not at all important	(●)	( )	( )	( )	( )	essential

Clear selection

Does your paper address subitem 5-x?

Copy and paste relevant sections from the manuscript (include quotes in quotation marks "like this" to indicate direct quotes from your manuscript), or elaborate on this item by providing additional information not in the ms, or briefly explain why the item is not applicable/relevant for your study

NA – There was no human involvement

5-xi) Report any prompts/reminders used

Report any prompts/reminders used: Clarify if there were prompts (letters, emails, phone calls, SMS) to use the application, what triggered them, frequency etc. It may be necessary to distinguish between the level of prompts/reminders required for the trial, and the level of prompts/reminders for a routine application outside of a RCT setting (discuss under item 21 – generalizability).

	1	2	3	4	5	
subitem not at all important	○	○	○	◉	○	essential

Clear selection

Does your paper address subitem 5-xi? *

Copy and paste relevant sections from the manuscript (include quotes in quotation marks "like this" to indicate direct quotes from your manuscript), or elaborate on this item by providing additional information not in the ms, or briefly explain why the item is not applicable/relevant for your study

"To improve adherence participants using the GIA app received daily notifications via the app as well as a weekly reminder via email."

5-xii) Describe any co-interventions (incl. training/support)

Describe any co-interventions (incl. training/support): Clearly state any interventions that are provided in addition to the targeted eHealth intervention, as ehealth intervention may not be designed as stand-alone intervention. This includes training sessions and support [1]. It may be necessary to distinguish between the level of training required for the trial, and the level of training for a routine application outside of a RCT setting (discuss under item 21 – generalizability.

	1	2	3	4	5	
subitem not at all important	(●)	○	○	○	○	essential

Clear selection

Does your paper address subitem 5-xii? *

Copy and paste relevant sections from the manuscript (include quotes in quotation marks "like this" to indicate direct quotes from your manuscript), or elaborate on this item by providing additional information not in the ms, or briefly explain why the item is not applicable/relevant for your study

NA – There were no co-interventions in addition to the targeted eHealth intervention.

6a) Completely defined pre-specified primary and secondary outcome measures, including how and when they were assessed

Does your paper address CONSORT subitem 6a? *

Copy and paste relevant sections from the manuscript (include quotes in quotation marks "like this" to indicate direct quotes from your manuscript), or elaborate on this item by providing additional information not in the ms, or briefly explain why the item is not applicable/relevant for your study

"Participant's depression, anxiety, and stress levels were measured with the Depression, Anxiety, and Stress Scale (DASS-21) [30], which is a set of three self-report scales each consisting of seven items to measure the individual's emotional states of depression ("I couldn't seem to experience any positive feeling at all"), anxiety ("I felt I was close to panic"), and stress ("I found it difficult to relax"). The DASS-21 has shown good psychometric properties, with strong internal consistency in both total scoring and scale set scoring, and has been found to have adequate construct validity. In the current sample, the reliability of DASS-21 was good with Cronbach's alpha values of 0.89, 0.82 and 0.76 for the subscales of depression, anxiety and stress respectively.

Negative affectivity and social inhibition were measured with the Type D Personality Scale (DS14). The DS14 has shown good internal consistency for both negative affectivity and social inhibition. In the current sample, the reliability of DS14 was good with Cronbach's alpha values of 0.84 0.86 for the subscales of negative affectivity and social inhibition, respectively. All measures were completed online."

6a-i) Online questionnaires: describe if they were validated for online use and apply CHERRIES items to describe how the questionnaires were designed/deployed

If outcomes were obtained through online questionnaires, describe if they were validated for online use and apply CHERRIES items to describe how the questionnaires were designed/deployed [9].

	1	2	3	4	5	
subitem not at all important	○	○	◉	○	○	essential

Clear selection

Does your paper address subitem 6a-i?

Copy and paste relevant sections from manuscript text

All assessments used have been validated and used in online studies in previous research. Response rates of individuals were measured, prevention of multiple entries from the same individual was also monitored. Questionnaires were approved by an IRB prior to use in the study.

6a-ii) Describe whether and how "use" (including intensity of use/dosage) was defined/measured/monitored

Describe whether and how "use" (including intensity of use/dosage) was defined/measured/monitored (logins, logfile analysis, etc.). Use/adoption metrics are important process outcomes that should be reported in any ehealth trial.

	1	2	3	4	5	
subitem not at all important	○	○	○	◉	○	essential

Clear selection

Does your paper address subitem 6a-ii?

Copy and paste relevant sections from manuscript text

"PROSIT and GIA app usage (i.e., registration date, frequency of accessing the app, date of last visit) were logged."

6a-iii) Describe whether, how, and when qualitative feedback from participants was obtained

Describe whether, how, and when qualitative feedback from participants was obtained (e.g., through emails, feedback forms, interviews, focus groups).

	1	2	3	4	5	
subitem not at all important	(●)	( )	( )	( )	( )	essential

Clear selection

Does your paper address subitem 6a-iii?

Copy and paste relevant sections from manuscript text

NA - there was not qualitative feedback from participants

6b) Any changes to trial outcomes after the trial commenced, with reasons

Does your paper address CONSORT subitem 6b? *

Copy and paste relevant sections from the manuscript (include quotes in quotation marks "like this" to indicate direct quotes from your manuscript), or elaborate on this item by providing additional information not in the ms, or briefly explain why the item is not applicable/relevant for your study

NA – No changes to trial outcomes occurred after the trial commenced

7a) How sample size was determined

NPT: When applicable, details of whether and how the clustering by care provides or centers was addressed

7a-i) Describe whether and how expected attrition was taken into account when calculating the sample size

Describe whether and how expected attrition was taken into account when calculating the sample size.

	1	2	3	4	5	
subitem not at all important	○	○	○	○	◉	essential

Clear selection

Does your paper address subitem 7a-i?

Copy and paste relevant sections from manuscript title (include quotes in quotation marks "like this" to indicate direct quotes from your manuscript), or elaborate on this item by providing additional information not in the ms, or briefly explain why the item is not applicable/relevant for your study

“Anticipating approximately 30% attrition, we aimed to enroll a minimum of 120 participants.”“Anticipating approximately 30% attrition, we aimed to enroll a minimum of 120 participants."

7b) When applicable, explanation of any interim analyses and stopping guidelines

Does your paper address CONSORT subitem 7b? *

Copy and paste relevant sections from the manuscript (include quotes in quotation marks "like this" to indicate direct quotes from your manuscript), or elaborate on this item by providing additional information not in the ms, or briefly explain why the item is not applicable/relevant for your study

NA – There were no interim analyses or stopping guidelines.

8a) Method used to generate the random allocation sequence

NPT: When applicable, how care providers were allocated to each trial group

Does your paper address CONSORT subitem 8a? *

Copy and paste relevant sections from the manuscript (include quotes in quotation marks "like this" to indicate direct quotes from your manuscript), or elaborate on this item by providing additional information not in the ms, or briefly explain why the item is not applicable/relevant for your study

"Participants were randomized with a 1:1 allocation in blocks of 4 using a computer-generated algorithm. Randomization was conducted by a researcher who was not otherwise involved in the study. Data analysts had no interactions with participants."

8b) Type of randomisation; details of any restriction (such as blocking and block size)

Does your paper address CONSORT subitem 8b? *

Copy and paste relevant sections from the manuscript (include quotes in quotation marks "like this" to indicate direct quotes from your manuscript), or elaborate on this item by providing additional information not in the ms, or briefly explain why the item is not applicable/relevant for your study

"1:1 allocation in blocks of 4 using a computer-generated algorithm."

9) Mechanism used to implement the random allocation sequence (such as sequentially numbered containers), describing any steps taken to conceal the sequence until interventions were assigned

Does your paper address CONSORT subitem 9? *

Copy and paste relevant sections from the manuscript (include quotes in quotation marks "like this" to indicate direct quotes from your manuscript), or elaborate on this item by providing additional information not in the ms, or briefly explain why the item is not applicable/relevant for your study

"Randomization was conducted by a researcher who was not otherwise involved in the study. Data analysts had no interactions with participants."

10) Who generated the random allocation sequence, who enrolled participants, and who assigned participants to interventions

Does your paper address CONSORT subitem 10? *

Copy and paste relevant sections from the manuscript (include quotes in quotation marks "like this" to indicate direct quotes from your manuscript), or elaborate on this item by providing additional information not in the ms, or briefly explain why the item is not applicable/relevant for your study

"Randomization was conducted by a researcher who was not otherwise involved in the study. Data analysts had no interactions with participants"

11a) If done, who was blinded after assignment to interventions (for example, participants, care providers, those assessing outcomes) and how

NPT: Whether or not administering co-interventions were blinded to group assignment

11a-i) Specify who was blinded, and who wasn't

Specify who was blinded, and who wasn't. Usually, in web-based trials it is not possible to blind the participants [1, 3] (this should be clearly acknowledged), but it may be possible to blind outcome assessors, those doing data analysis or those administering co-interventions (if any).

	1	2	3	4	5	
subitem not at all important	○	○	○	○	◉	essential

Clear selection

Does your paper address subitem 11a-i? *

Copy and paste relevant sections from the manuscript (include quotes in quotation marks "like this" to indicate direct quotes from your manuscript), or elaborate on this item by providing additional information not in the ms, or briefly explain why the item is not applicable/relevant for your study

It is not possible to blind participants in this study. Participants likely can guess which group they are part of it because one is just mobile sensing and GIA is additional. They can infer this based one the consent form cannot double blind them.

11a-ii) Discuss e.g., whether participants knew which intervention was the "intervention of interest" and which one was the "comparator"

Informed consent procedures (4a-ii) can create biases and certain expectations - discuss e.g., whether participants knew which intervention was the "intervention of interest" and which one was the "comparator".

	1	2	3	4	5	
subitem not at all important	○	○	○	○	◉	essential

Clear selection

Does your paper address subitem 11a-ii?

Copy and paste relevant sections from the manuscript (include quotes in quotation marks "like this" to indicate direct quotes from your manuscript), or elaborate on this item by providing additional information not in the ms, or briefly explain why the item is not applicable/relevant for your study

Participants can guess which group they are in based on the consent form.

11b) If relevant, description of the similarity of interventions
(this item is usually not relevant for ehealth trials as it refers to similarity of a placebo or sham intervention to a active medication/intervention)

Does your paper address CONSORT subitem 11b? *

Copy and paste relevant sections from the manuscript (include quotes in quotation marks "like this" to indicate direct quotes from your manuscript), or elaborate on this item by providing additional information not in the ms, or briefly explain why the item is not applicable/relevant for your study

NA – There were no similarity of interventions

12a) Statistical methods used to compare groups for primary and secondary outcomes
NPT: When applicable, details of whether and how the clustering by care providers or centers was addressed

Does your paper address CONSORT subitem 12a? *

Copy and paste relevant sections from the manuscript (include quotes in quotation marks "like this" to indicate direct quotes from your manuscript), or elaborate on this item by providing additional information not in the ms, or briefly explain why the item is not applicable/relevant for your study

NA - There were no secondary outcomes

12a-i) Imputation techniques to deal with attrition / missing values

Imputation techniques to deal with attrition / missing values: Not all participants will use the intervention/comparator as intended and attrition is typically high in ehealth trials. Specify how participants who did not use the application or dropped out from the trial were treated in the statistical analysis (a complete case analysis is strongly discouraged, and simple imputation techniques such as LOCF may also be problematic [4]).

	1	2	3	4	5	
subitem not at all important	◉	○	○	○	○	essential

Clear selection

Does your paper address subitem 12a-i? *

Copy and paste relevant sections from the manuscript (include quotes in quotation marks "like this" to indicate direct quotes from your manuscript), or elaborate on this item by providing additional information not in the ms, or briefly explain why the item is not applicable/relevant for your study

“Missing data was handled using maximum likelihood estimation.”

12b) Methods for additional analyses, such as subgroup analyses and adjusted analyses

Does your paper address CONSORT subitem 12b? *

Copy and paste relevant sections from the manuscript (include quotes in quotation marks "like this" to indicate direct quotes from your manuscript), or elaborate on this item by providing additional information not in the ms, or briefly explain why the item is not applicable/relevant for your study

"Descriptive statistics were computed for demographics and outcome measures at baseline in the full sample of students and a subsample of those who showed at least moderate symptomatology."

"Of those participants randomized to the gratitude intervention, 44 (77.2%) started and used the app in the full sample and 21 (67.7%) in the subsample."

"In the subsample 11 (35.5%) gratitude intervention participants and 6 (24.0%) control participants were lost. These differences were not statistically significant (p = 0.59 in the full sample and p = 0.35 in the subsample)."

"Participants in the subsample showing at least moderate symptomatology used the app slightly less, which was not statistically significant p =0.34; specifically, 21 participants (68%) accessed the app on average 16 times (SD = 11.27) over 9 days (SD = 7.73)."

"The results indicated a statistically significant effect (i.e., time * group interaction) for negative affectivity (d = -0.68, 95% CI -1.34 – -0.01, p = 0.046) but not social inhibition (d = -0.61, 95% CI -1.27 – 0.05, p = 0.07) in the subsample showing at least moderate symptomatology."

X26) REB/IRB Approval and Ethical Considerations [recommended as subheading under "Methods"] (not a CONSORT item)

X26-i) Comment on ethics committee approval

	1	2	3	4	5	
subitem not at all important	○	○	○	○	◉	essential

Clear selection

Does your paper address subitem X26-i?

Copy and paste relevant sections from the manuscript (include quotes in quotation marks "like this" to indicate direct quotes from your manuscript), or elaborate on this item by providing additional information not in the ms, or briefly explain why the item is not applicable/relevant for your study

"This study was approved by the Dalhousie University Research Ethics Board (REB 2021-5460)."

x26-ii) Outline informed consent procedures

Outline informed consent procedures e.g., if consent was obtained offline or online (how? Checkbox, etc.?), and what information was provided (see 4a-ii). See [6] for some items to be included in informed consent documents.

	1	2	3	4	5	
subitem not at all important	○	○	○	○	◉	essential

Clear selection

Does your paper address subitem X26-ii?

Copy and paste relevant sections from the manuscript (include quotes in quotation marks "like this" to indicate direct quotes from your manuscript), or elaborate on this item by providing additional information not in the ms, or briefly explain why the item is not applicable/relevant for your study

this was obtained online via REDCap

X26-iii) Safety and security procedures

Safety and security procedures, incl. privacy considerations, and any steps taken to reduce the likelihood or detection of harm (e.g., education and training, availability of a hotline)

	1	2	3	4	5	
subitem not at all important	(•)	( )	( )	( )	( )	essential

Clear selection

Does your paper address subitem X26-iii?

Copy and paste relevant sections from the manuscript (include quotes in quotation marks "like this" to indicate direct quotes from your manuscript), or elaborate on this item by providing additional information not in the ms, or briefly explain why the item is not applicable/relevant for your study

We did not have any education, training, hotline availability etc.

RESULTS

13a) For each group, the numbers of participants who were randomly assigned, received intended treatment, and were analysed for the primary outcome

NPT: The number of care providers or centers performing the intervention in each group and the number of patients treated by each care provider in each center

Does your paper address CONSORT subitem 13a? *

Copy and paste relevant sections from the manuscript (include quotes in quotation marks "like this" to indicate direct quotes from your manuscript), or elaborate on this item by providing additional information not in the ms, or briefly explain why the item is not applicable/relevant for your study

"Over 137 individuals completed the eligibility screener for the study. Out of the 137 who met the inclusion criteria, 120 (87.6%) were enrolled and completed the baseline assessment. In the full sample, 57 participants were randomized to the gratitude intervention and 63 to the control group of which 31 and 25 displayed at least moderately significant symptomatology. Of those participants randomized to the gratitude intervention, 44 (77.2%) started and used the app in the full sample and 21 (67.7%) in the subsample. Follow-ups were completed 3 weeks later. In the full sample, 16 (28.1%) gratitude intervention and 15 (23.8%) control participants were lost to follow-up. In the subsample 11 (35.5%) gratitude intervention participants and 6 (24.0%) control participants were lost."

13b) For each group, losses and exclusions after randomisation, together with reasons

Does your paper address CONSORT subitem 13b? (NOTE: Preferably, this is shown in a CONSORT flow diagram) *

Copy and paste relevant sections from the manuscript (include quotes in quotation marks "like this" to indicate direct quotes from your manuscript), or elaborate on this item by providing additional information not in the ms, or briefly explain why the item is not applicable/relevant for your study

"Out of the 137 who met the inclusion criteria, 120 (87.6%) were enrolled and completed the baseline assessment."

13b-i) Attrition diagram

Strongly recommended: An attrition diagram (e.g., proportion of participants still logging in or using the intervention/comparator in each group plotted over time, similar to a survival curve) or other figures or tables demonstrating usage/dose/engagement.

	1	2	3	4	5	
subitem not at all important	(selected)	○	○	○	○	essential

Clear selection

Does your paper address subitem 13b-i?

Copy and paste relevant sections from the manuscript or cite the figure number if applicable (include quotes in quotation marks "like this" to indicate direct quotes from your manuscript), or elaborate on this item by providing additional information not in the ms, or briefly explain why the item is not applicable/relevant for your study

NA - We do not have this data therefore there is no attrition diagram.

14a) Dates defining the periods of recruitment and follow-up

Does your paper address CONSORT subitem 14a? *

Copy and paste relevant sections from the manuscript (include quotes in quotation marks "like this" to indicate direct quotes from your manuscript), or elaborate on this item by providing additional information not in the ms, or briefly explain why the item is not applicable/relevant for your study

"Recruitment ran from November 11, 2021, to March 15, 2023"
"Follow-ups were completed 3 weeks later."

14a-i) Indicate if critical “secular events” fell into the study period

Indicate if critical “secular events” fell into the study period, e.g., significant changes in Internet resources available or “changes in computer hardware or Internet delivery resources”

	1	2	3	4	5	
subitem not at all important	(●)	( )	( )	( )	( )	essential

Clear selection

Does your paper address subitem 14a-i?

Copy and paste relevant sections from the manuscript (include quotes in quotation marks "like this" to indicate direct quotes from your manuscript), or elaborate on this item by providing additional information not in the ms, or briefly explain why the item is not applicable/relevant for your study

NA – No critical secular events fell into the study period

14b) Why the trial ended or was stopped (early)

Does your paper address CONSORT subitem 14b? *

Copy and paste relevant sections from the manuscript (include quotes in quotation marks "like this" to indicate direct quotes from your manuscript), or elaborate on this item by providing additional information not in the ms, or briefly explain why the item is not applicable/relevant for your study

NA – The trial was not ended or stopped early

15) A table showing baseline demographic and clinical characteristics for each group

NPT: When applicable, a description of care providers (case volume, qualification, expertise, etc.) and centers (volume) in each group

Does your paper address CONSORT subitem 15? *

Copy and paste relevant sections from the manuscript (include quotes in quotation marks "like this" to indicate direct quotes from your manuscript), or elaborate on this item by providing additional information not in the ms, or briefly explain why the item is not applicable/relevant for your study

This is included in the manscript

15-i) Report demographics associated with digital divide issues

In ehealth trials it is particularly important to report demographics associated with digital divide issues, such as age, education, gender, social-economic status, computer/Internet/ehealth literacy of the participants, if known.

	1	2	3	4	5	
subitem not at all important	○	○	○	◉	○	essential

Clear selection

Does your paper address subitem 15-i? *

Copy and paste relevant sections from the manuscript (include quotes in quotation marks "like this" to indicate direct quotes from your manuscript), or elaborate on this item by providing additional information not in the ms, or briefly explain why the item is not applicable/relevant for your study

"Out of the 137 who met the inclusion criteria, 120 (87.6%) were enrolled and completed the baseline assessment. In the full sample, 57 participants were randomized to the gratitude intervention and 63 to the control group of which 31 and 25 displayed at least moderately significant symptomatology. Of those participants randomized to the gratitude intervention, 44 (77.2%) started and used the app in the full sample and 21 (67.7%) in the subsample. Follow-ups were completed 3 weeks later. In the full sample, 16 (28.1%) gratitude intervention and 15 (23.8%) control participants were lost to follow-up. In the subsample 11 (35.5%) gratitude intervention participants and 6 (24.0%) control participants were lost. These differences were not statistically significant (p = 0.59 in the full sample and p = 0.35 in the subsample)."

16) For each group, number of participants (denominator) included in each analysis and whether the analysis was by original assigned groups

16-i) Report multiple “denominators” and provide definitions

Report multiple “denominators” and provide definitions: Report N’s (and effect sizes) “across a range of study participation [and use] thresholds” [1], e.g., N exposed, N consented, N used more than x times, N used more than y weeks, N participants “used” the intervention/comparator at specific pre-defined time points of interest (in absolute and relative numbers per group). Always clearly define “use” of the intervention.

	1	2	3	4	5	
subitem not at all important	○	○	○	◉	○	essential

Clear selection

Does your paper address subitem 16-i? *

Copy and paste relevant sections from the manuscript (include quotes in quotation marks "like this" to indicate direct quotes from your manuscript), or elaborate on this item by providing additional information not in the ms, or briefly explain why the item is not applicable/relevant for your study

"Log data showed that most participants (n = 44, 77%) in the full sample randomized to the gratitude intervention group accessed the app. Participants opened the app on average 18 times (SD = 11.04) over 9 days (SD = 6.90). Participants in the subsample showing at least moderate symptomatology used the app slightly less, which was not statistically significant p =0.34; specifically, 21 participants (68%) accessed the app on average 16 times (SD = 11.27) over 9 days (SD = 7.73)."

16-ii) Primary analysis should be intent-to-treat

Primary analysis should be intent-to-treat, secondary analyses could include comparing only “users”, with the appropriate caveats that this is no longer a randomized sample (see 18-i).

	1	2	3	4	5	
subitem not at all important	○	○	○	○	◉	essential

Clear selection

Does your paper address subitem 16-ii?

Copy and paste relevant sections from the manuscript (include quotes in quotation marks "like this" to indicate direct quotes from your manuscript), or elaborate on this item by providing additional information not in the ms, or briefly explain why the item is not applicable/relevant for your study

"An intent-to-treat (ITT) approach was used, consistent with CONSORT guidelines (i.e., all participants who were randomized to receive enrollment information for the intervention were included in analyses) [33]."

17a) For each primary and secondary outcome, results for each group, and the estimated effect size and its precision (such as 95% confidence interval)

Does your paper address CONSORT subitem 17a? *

Copy and paste relevant sections from the manuscript (include quotes in quotation marks "like this" to indicate direct quotes from your manuscript), or elaborate on this item by providing additional information not in the ms, or briefly explain why the item is not applicable/relevant for your study

""The results indicated a statistically significant medium treatment effect (i.e., time * group interaction) for mental health symptoms (Cohen’s d = -0.68, 95% CI -1.34 – -0.03, p = 0.04)) in the subsample showing at least moderate symptomatology but not the full sample (d = 0.16, 95%CI -0.26 - 0.58, P = .46)"
"The results indicated a statistically significant effect (i.e., time * group interaction) for negative affectivity (d = -0.68, 95% CI -1.34 – -0.01, p = 0.046) but not social inhibition (d = -0.61, 95% CI -1.27 – 0.05, p = 0.07) in the subsample showing at least moderate symptomatology. The gratitude intervention group scored significantly lower on negative affectivity and trend-wise for social inhibition. No effects were observed in the full sample (negative affectivity d = -0.18, 95% CI -0.60 - 0.24, P = .41; social inhibition d = -0.18, 95% CI -0.60 - 0.24, P = .41). No differences in technology engagement as measured by the PROSIT app were observed in the gratitude intervention group compared to the control group in the subsample with at least moderate symptomatology (t=0.56. p = 0.58) or the full sample (t=1.20. p = 0.23)."

17a-i) Presentation of process outcomes such as metrics of use and intensity of use

In addition to primary/secondary (clinical) outcomes, the presentation of process outcomes such as metrics of use and intensity of use (dose, exposure) and their operational definitions is critical. This does not only refer to metrics of attrition (13-b) (often a binary variable), but also to more continuous exposure metrics such as "average session length". These must be accompanied by a technical description how a metric like a "session" is defined (e.g., timeout after idle time) [1] (report under item 6a).

	1	2	3	4	5	
subitem not at all important	○	○	○	◉	○	essential

Clear selection

Does your paper address subitem 17a-i?

Copy and paste relevant sections from the manuscript (include quotes in quotation marks "like this" to indicate direct quotes from your manuscript), or elaborate on this item by providing additional information not in the ms, or briefly explain why the item is not applicable/relevant for your study

"Participants opened the app on average 18 times (SD = 11.04) over 9 days (SD = 6.90)."

17b) For binary outcomes, presentation of both absolute and relative effect sizes is recommended

Does your paper address CONSORT subitem 17b? *

Copy and paste relevant sections from the manuscript (include quotes in quotation marks "like this" to indicate direct quotes from your manuscript), or elaborate on this item by providing additional information not in the ms, or briefly explain why the item is not applicable/relevant for your study

NA - There were no binary outcomes

18) Results of any other analyses performed, including subgroup analyses and adjusted analyses, distinguishing pre-specified from exploratory

Does your paper address CONSORT subitem 18? *

Copy and paste relevant sections from the manuscript (include quotes in quotation marks "like this" to indicate direct quotes from your manuscript), or elaborate on this item by providing additional information not in the ms, or briefly explain why the item is not applicable/relevant for your study

"In the subsample, the gratitude intervention group scored significantly lower on depression, anxiety, and stress as measured by the DASS-21. The number of times the app was used was not correlated with changes in clinical symptomatology in the subsample with at least moderate symptomatology (r=0.01, p = 0.98) or the full sample (r=-0.04 p = 0.79)."

18-i) Subgroup analysis of comparing only users

A subgroup analysis of comparing only users is not uncommon in ehealth trials, but if done, it must be stressed that this is a self-selected sample and no longer an unbiased sample from a randomized trial (see 16-iii).

	1	2	3	4	5	
subitem not at all important	○	○	○	○	●	essential

Clear selection

Does your paper address subitem 18-i?

Copy and paste relevant sections from the manuscript (include quotes in quotation marks "like this" to indicate direct quotes from your manuscript), or elaborate on this item by providing additional information not in the ms, or briefly explain why the item is not applicable/relevant for your study

“The results indicated a statistically significant medium treatment effect (i.e., time * group interaction) for mental health symptoms (Cohen’s d = -0.68, 95% CI -1.34 – -0.03, p = 0.04)) in the subsample showing at least moderate symptomatology but not the full sample (d = 0.16, 95%CI -0.26 - 0.58, P = .46). In the subsample, the gratitude intervention group scored significantly lower on depression, anxiety, and stress as measured by the DASS-21.”

19) All important harms or unintended effects in each group

(for specific guidance see CONSORT for harms)

Does your paper address CONSORT subitem 19? *

Copy and paste relevant sections from the manuscript (include quotes in quotation marks "like this" to indicate direct quotes from your manuscript), or elaborate on this item by providing additional information not in the ms, or briefly explain why the item is not applicable/relevant for your study

NA - No important harms or unintended effects occurred.

19-i) Include privacy breaches, technical problems

Include privacy breaches, technical problems. This does not only include physical “harm” to participants, but also incidents such as perceived or real privacy breaches [1], technical problems, and other unexpected/unintended incidents. “Unintended effects” also includes unintended positive effects [2].

	1	2	3	4	5	
subitem not at all important	(•)	( )	( )	( )	( )	essential

Clear selection

Does your paper address subitem 19-i?

Copy and paste relevant sections from the manuscript (include quotes in quotation marks "like this" to indicate direct quotes from your manuscript), or elaborate on this item by providing additional information not in the ms, or briefly explain why the item is not applicable/relevant for your study

NA - No privacy breaches or technical problems.

19-ii) Include qualitative feedback from participants or observations from staff/researchers

Include qualitative feedback from participants or observations from staff/researchers, if available, on strengths and shortcomings of the application, especially if they point to unintended/unexpected effects or uses. This includes (if available) reasons for why people did or did not use the application as intended by the developers.

	1	2	3	4	5	
subitem not at all important	(●)	( )	( )	( )	( )	essential

Clear selection

Does your paper address subitem 19-ii?

Copy and paste relevant sections from the manuscript (include quotes in quotation marks "like this" to indicate direct quotes from your manuscript), or elaborate on this item by providing additional information not in the ms, or briefly explain why the item is not applicable/relevant for your study

NA, we did not collect and do not have this data.

DISCUSSION

22) Interpretation consistent with results, balancing benefits and harms, and considering other relevant evidence

NPT: In addition, take into account the choice of the comparator, lack of or partial blinding, and unequal expertise of care providers or centers in each group

22-i) Restate study questions and summarize the answers suggested by the data, starting with primary outcomes and process outcomes (use)

Restate study questions and summarize the answers suggested by the data, starting with primary outcomes and process outcomes (use).

	1	2	3	4	5	
subitem not at all important	○	○	○	◉	○	essential

Clear selection

Does your paper address subitem 22-i? *

Copy and paste relevant sections from the manuscript (include quotes in quotation marks "like this" to indicate direct quotes from your manuscript), or elaborate on this item by providing additional information not in the ms, or briefly explain why the item is not applicable/relevant for your study

"The results indicated a statistically significant medium treatment effect (i.e., time * group interaction) for mental health symptoms (Cohen's d = -0.68, 95% CI -1.34 – -0.03, p = 0.04)) in the subsample showing at least moderate symptomatology but not the full sample (d = 0.16, 95%CI -0.26 - 0.58, P = .46)"
"The results indicated a statistically significant effect (i.e., time * group interaction) for negative affectivity (d = -0.68, 95% CI -1.34 – -0.01, p = 0.046) but not social inhibition (d = -0.61, 95% CI -1.27 – 0.05, p = 0.07) in the subsample showing at least moderate symptomatology. The gratitude intervention group scored significantly lower on negative affectivity and trend-wise for social inhibition. No effects were observed in the full sample (negative affectivity d = -0.18, 95% CI -0.60 - 0.24, P = .41; social inhibition d = -0.18, 95% CI -0.60 - 0.24, P = .41). No differences in technology engagement as measured by the PROSIT app were observed in the gratitude intervention group compared to the control group in the subsample with at least moderate symptomatology (t=0.56. p = 0.58) or the full sample (t=1.20. p = 0.23)."

22-ii) Highlight unanswered new questions, suggest future research

Highlight unanswered new questions, suggest future research.

	1	2	3	4	5	
subitem not at all important	○	○	○	○	◉	essential

Clear selection

Does your paper address subitem 22-ii?

Copy and paste relevant sections from the manuscript (include quotes in quotation marks "like this" to indicate direct quotes from your manuscript), or elaborate on this item by providing additional information not in the ms, or briefly explain why the item is not applicable/relevant for your study

"Future research should assess and test even more proximal mediators in serial multiple mediation models to further understand how the gratitude intervention works."
"Future studies should examine this intervention’s effectiveness in other populations and explore the unique impacts of different gratitude exercise modalities."
"While the current study did not include participants already receiving mental health treatment, future studies, interventions, and mental health treatments could look to incorporating gratitude interventions with other mental health treatments."

20) Trial limitations, addressing sources of potential bias, imprecision, and, if relevant, multiplicity of analyses

20-i) Typical limitations in ehealth trials

Typical limitations in ehealth trials: Participants in ehealth trials are rarely blinded. Ehealth trials often look at a multiplicity of outcomes, increasing risk for a Type I error. Discuss biases due to non-use of the intervention/usability issues, biases through informed consent procedures, unexpected events.

	1	2	3	4	5	
subitem not at all important	○	○	○	○	◉	essential

Clear selection

Does your paper address subitem 20-i? *

Copy and paste relevant sections from the manuscript (include quotes in quotation marks "like this" to indicate direct quotes from your manuscript), or elaborate on this item by providing additional information not in the ms, or briefly explain why the item is not applicable/relevant for your study

"Second, study dropout in the gratitude intervention group was relatively high and somewhat higher than in the control group. While this is a common finding in online intervention studies in university student populations (intervention group completion rates: 43–66%), this might limit the interpretation of results [45-47]. Reported trends could be partially related to specific participant characteristics (e.g., having a stronger motivation for gratitude interventions) by the participants in the sample that adhered to the intervention. In addition, the app format was not compared to other forms of delivery and the effects of certain app features or persuasive elements were not tested. Further experiments are needed to unravel the specific effects of delivery features [48]. Third, there was no long-term follow-up on well-being outcomes after the intervention and follow-up only occurred immediately post-intervention. Therefore, we are unable to conclude the longer-term effects of mobile gratitude interventions in this population. Fourth, we investigated the gratitude intervention's effect on the potential mechanisms of negative affectivity and social inhibition. Yet the current study didn't have the power to examine them as mediators."

21) Generalisability (external validity, applicability) of the trial findings

NPT: External validity of the trial findings according to the intervention, comparators, patients, and care providers or centers involved in the trial

21-i) Generalizability to other populations

Generalizability to other populations: In particular, discuss generalizability to a general Internet population, outside of a RCT setting, and general patient population, including applicability of the study results for other organizations

	1	2	3	4	5	
subitem not at all important	○	○	○	○	◉	essential

Clear selection

Does your paper address subitem 21-i?

Copy and paste relevant sections from the manuscript (include quotes in quotation marks "like this" to indicate direct quotes from your manuscript), or elaborate on this item by providing additional information not in the ms, or briefly explain why the item is not applicable/relevant for your study

"This study had several limitations. Firstly, the study had a small sample size with a relatively homogenous sample population. The participants were predominantly Canadian, and female, and all were students. Thus, the study findings might not be generalizable to males."

21-ii) Discuss if there were elements in the RCT that would be different in a routine application setting

Discuss if there were elements in the RCT that would be different in a routine application setting (e.g., prompts/reminders, more human involvement, training sessions or other co-interventions) and what impact the omission of these elements could have on use, adoption, or outcomes if the intervention is applied outside of a RCT setting.

	1	2	3	4	5	
subitem not at all important	◉	○	○	○	○	essential

Clear selection

Does your paper address subitem 21-ii?

Copy and paste relevant sections from the manuscript (include quotes in quotation marks "like this" to indicate direct quotes from your manuscript), or elaborate on this item by providing additional information not in the ms, or briefly explain why the item is not applicable/relevant for your study

NA – There were no elements in the RCT that would be different in a routine application setting.

OTHER INFORMATION

23) Registration number and name of trial registry

Does your paper address CONSORT subitem 23? *

Copy and paste relevant sections from the manuscript (include quotes in quotation marks "like this" to indicate direct quotes from your manuscript), or elaborate on this item by providing additional information not in the ms, or briefly explain why the item is not applicable/relevant for your study

NCT06621745, A Gratitude Intervention App to Reduce Symptoms of Depression, Anxiety and Stress

24) Where the full trial protocol can be accessed, if available

Does your paper address CONSORT subitem 24? *

Cite a Multimedia Appendix, other reference, or copy and paste relevant sections from the manuscript (include quotes in quotation marks "like this" to indicate direct quotes from your manuscript), or elaborate on this item by providing additional information not in the ms, or briefly explain why the item is not applicable/relevant for your study

https://clinicaltrials.gov/study/NCT06621745

25) Sources of funding and other support (such as supply of drugs), role of funders

Does your paper address CONSORT subitem 25? *

Copy and paste relevant sections from the manuscript (include quotes in quotation marks "like this" to indicate direct quotes from your manuscript), or elaborate on this item by providing additional information not in the ms, or briefly explain why the item is not applicable/relevant for your study

"This work was partly funded by the Canadian Institute for Health Research (CIHR) Canadian Research Chairs (CRC) stipend [Award number 1024586]; the Canadian Foundation for Innovation (CFI) John R. Evans Leaders Fund (JELF) [Grant number 38835]. The funding sources had no role in study design, data collection, data analysis, data interpretation, the writing of the manuscript or the decision to (and where to) submit the article for publication."

X27) Conflicts of Interest (not a CONSORT item)

X27-i) State the relation of the study team towards the system being evaluated

In addition to the usual declaration of interests (financial or otherwise), also state the relation of the study team towards the system being evaluated, i.e., state if the authors/evaluators are distinct from or identical with the developers/sponsors of the intervention.

	1	2	3	4	5	
subitem not at all important	○	○	○	●	○	essential

Clear selection

Does your paper address subitem X27-i?

Copy and paste relevant sections from the manuscript (include quotes in quotation marks "like this" to indicate direct quotes from your manuscript), or elaborate on this item by providing additional information not in the ms, or briefly explain why the item is not applicable/relevant for your study

"The authors declare that they have no known competing financial interests or personal relationships that could have appeared to influence the work reported in this paper."

About the CONSORT EHEALTH checklist

As a result of using this checklist, did you make changes in your manuscript? *

- ( ) yes, major changes
- (•) yes, minor changes
- ( ) no

What were the most important changes you made as a result of using this checklist?

Your answer

How much time did you spend on going through the checklist INCLUDING making changes in your manuscript *

Time spent editing this document was 8+ hours

As a result of using this checklist, do you think your manuscript has improved? *

- (•) yes
- ( ) no
- ( ) Other:

Would you like to become involved in the CONSORT EHEALTH group?

This would involve for example becoming involved in participating in a workshop and writing an "Explanation and Elaboration" document

- ( ) yes
- (•) no
- ( ) Other:

Clear selection

Any other comments or questions on CONSORT EHEALTH

Your answer

STOP - Save this form as PDF before you click submit

To generate a record that you filled in this form, we recommend to generate a PDF of this page (on a Mac, simply select "print" and then select "print as PDF") before you submit it.

When you submit your (revised) paper to JMIR, please upload the PDF as supplementary file.

Don't worry if some text in the textboxes is cut off, as we still have the complete information in our database. Thank you!

Final step: Click submit !

Click submit so we have your answers in our database!

Submit Clear form

Never submit passwords through Google Forms.

This content is neither created nor endorsed by Google. Report Abuse - Terms of Service - Privacy Policy

Google Forms
